# Genome mining of biosynthetic and chemotherapeutic gene clusters in *Streptomyces* bacteria

**DOI:** 10.1038/s41598-020-58904-9

**Published:** 2020-02-06

**Authors:** Kaitlyn C. Belknap, Cooper J. Park, Brian M. Barth, Cheryl P. Andam

**Affiliations:** University of New Hampshire, Department of Molecular, Cellular and Biomedical Sciences, Durham, NH 03824 USA

**Keywords:** Evolution, Microbiology

## Abstract

*Streptomyces* bacteria are known for their prolific production of secondary metabolites, many of which have been widely used in human medicine, agriculture and animal health. To guide the effective prioritization of specific biosynthetic gene clusters (BGCs) for drug development and targeting the most prolific producer strains, knowledge about phylogenetic relationships of *Streptomyces* species, genome-wide diversity and distribution patterns of BGCs is critical. We used genomic and phylogenetic methods to elucidate the diversity of major classes of BGCs in 1,110 publicly available *Streptomyces* genomes. Genome mining of *Streptomyces* reveals high diversity of BGCs and variable distribution patterns in the *Streptomyces* phylogeny, even among very closely related strains. The most common BGCs are non-ribosomal peptide synthetases, type 1 polyketide synthases, terpenes, and lantipeptides. We also found that numerous *Streptomyces* species harbor BGCs known to encode antitumor compounds. We observed that strains that are considered the same species can vary tremendously in the BGCs they carry, suggesting that strain-level genome sequencing can uncover high levels of BGC diversity and potentially useful derivatives of any one compound. These findings suggest that a strain-level strategy for exploring secondary metabolites for clinical use provides an alternative or complementary approach to discovering novel pharmaceutical compounds from microbes.

## Introduction

Members of the bacterial genus *Streptomyces* (phylum Actinobacteria) are best known as major bacterial producers of antibiotics and other useful compounds commonly used in human medicine, animal health and agriculture^1,2^. Beginning in the 1940s when microbiologist Selman Waksman discovered streptomycin (the first effective drug against tuberculosis) produced by the soil-dwelling *Streptomyces griseus*^3^, *Streptomyces* species have received enormous attention in microbial sampling efforts. In the 21^st^ century, majority of all antibiotics have been developed from secondary metabolites produced by *Streptomyces*^4^. However, the last few decades saw a steep decline in the development and introduction of new medically relevant drugs to the market. This decline is partly due to the repeated re-discovery of the same molecules in the same ecological niches (often soil) and the high costs associated with drug development^5^. The increasing public health burden caused by multidrug resistance and the continuing need to find new treatments against non-communicable (chronic) diseases means that the search for bioactive compounds with novel mechanisms of action or with new cellular targets is greater than ever.

Genome mining approaches, which often involve identifying the genes involved in secondary metabolite production, have revealed an unprecedented biosynthetic potential in many microbial species^6–9^. These genes encode for the enzymes involved in peptide assembly, regulation, resistance, and synthesis of a secondary metabolite, and are physically clustered into groups called biosynthetic gene clusters (BGCs)^10^. Recent studies involving mining of large-scale genomic datasets have highlighted the tremendous potential of discovering novel and potentially relevant compounds from microbes^9,11–13^, which can allay some of the challenges in antibiotic development today^14,15^. While it has been estimated that many more novel natural products remain to be uncovered and functionally characterized, particularly those from poorly studied ecological niches, it is uncertain what the true number is or whether ongoing drug discovery efforts are reaching BGC saturation^16^. Moreover, a genome-wide study of representative Actinobacteria showed that *Streptomyces* genomes possess 25–70 BGCs, much more than any other actinobacterial genera, but only a small fraction of these bioactive products are produced when strains are cultivated in the laboratory^6^. This suggests that the full inventory of the chemical weapons possessed by a single bacterial strain remains poorly studied. Today, with the cost-effective and rapid increase in the number of bacterial genomes sequenced, one challenge encountered in current drug discovery efforts is how to effectively prioritize those strains with the greatest ability to produce new compounds and understand the extent of biosynthetic potential that exists in nature. Hence, knowledge about evolutionary relationships, BGC diversity, and distribution patterns of BGCs is crucial.

In this study, we aim to determine the diversity of BGCs and their phylogenetic distribution among 1,110 genomes of *Streptomyces*, the largest BGC study of a single genus to date. Genome mining reveals the presence of hybrid BGCs, variable distribution of antitumor BGCs and inter-strain differences in BGC content that together expand the repertoire of secondary metabolites that are potentially encoded by individual *Streptomyces* strains. Genome mining of closely related taxa can therefore greatly facilitate the discovery of novel pharmaceuticals and untapped sources of chemotherapeutic agents. These findings also highlight the importance of strain-level drug discovery approaches, exploring multiple genomes of closely related strains, rather than focusing on one strain representative of the entire species.

## Results

### Widespread distribution and diversity of BGCs in *Streptomyces*

**Figure 1:**
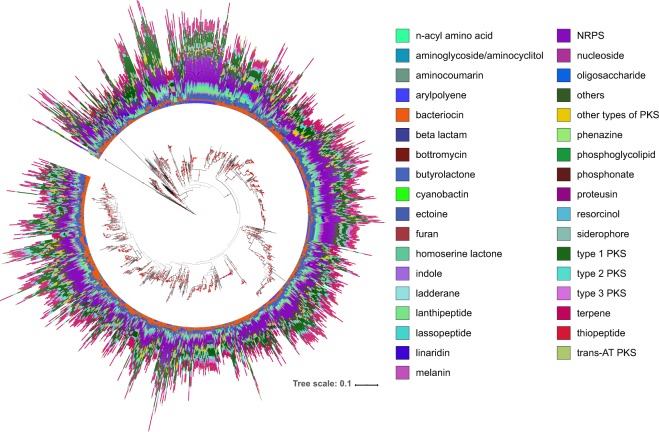
Phylogenetic distribution of the 34 major classes of BGCs in 1,107 *Streptomyces* genomes. The 35^th^ group of BGCs are those that contain a secondary metabolite-related protein but does not fit into any other category (labeled as “others”). BGCs were identified using antiSMASH. The midpoint-rooted maximum likelihood phylogenetic tree was calculated using sequence variation in the *rpoB* locus. Scale bar represents nucleotide substitutions per site. For visual clarity, only bootstrap values ≥70% are shown and are indicated by red dots. NRPS – non-ribosomal peptide synthetase, PKS – polyketide synthase, AT – acyltransferase. Members of each of two clusters labeled in blue and orange branches are those considered as belonging to the same species and are further examined in Fig. 4.

**Figure 2:**
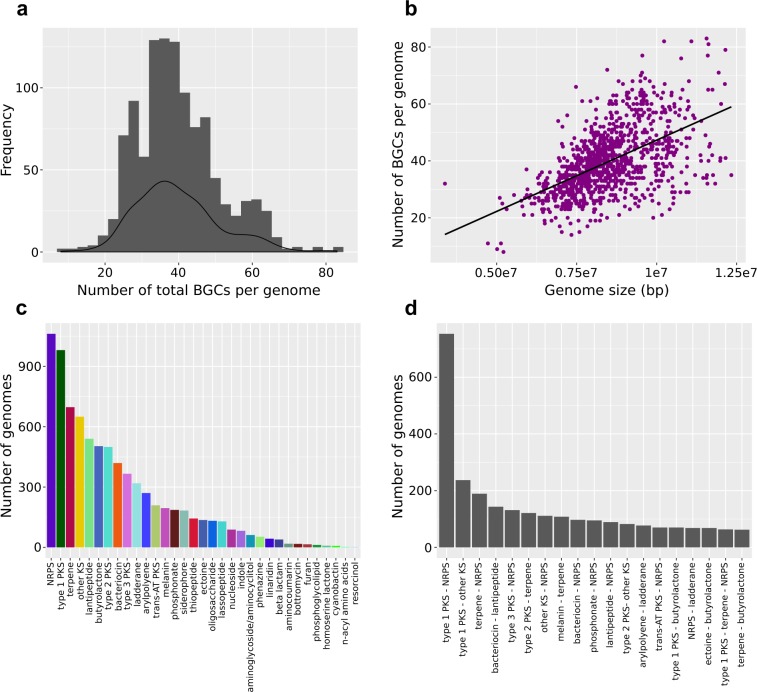
Frequency and diversity of BGCs. (**a**) Frequency distribution showing the number of total BGCs per genome. (**b**) Relationship between genome size and number of total BGCs per genome. (**c**) Frequency distribution showing the most common BGCs per genome. Because a genome can harbor multiple copies of the same BGC, we only show the total number of unique BGCs regardless of the number of copies there are in a single genome. (**d**) Frequency distribution showing the most common hybrid BGCs per genome. Only the total number of unique hybrid BGCs regardless of the number of copies there are of it present in a single genome.

We characterized the biosynthetic diversity in 1,110 *Streptomyces* genomes using antiSMASH^17^ (Supplementary Table S1). We detected a total of 34 major classes of BGCs, which is consistent with previous reports in other Actinobacteria genera such as *Salinispora* and *Amycolatopsis*^6,18,19^. Results show that *Streptomyces* bacteria carry between 8–83 BGCs per genome (mean = 39.64, s.d. = 11.40), with *Streptomyces rhizosphaericus* NRRL B-24304 (n = 83 BGCs), *Streptomyces* sp. NRRL B-1347 (n = 82 BGCs), *Streptomyces* sp. PRh5 (n = 82 BGCs), *Streptomyces milbemycinicus* NRRL 5739 (n = 81 BGCs) and *Streptomyces* sp. NBS 14/10 (n = 79 BGCs) having the highest number of BGCs (Figs. 1 and 2a). Genomes with the least number of BGCs include *Streptomyces gilvigriseus* MUSC 26 (n = 8 BGCs), *Streptomyces thermoautotrophicus* H1 (n = 9 BGCs) and *S. thermoautotrophicus* UBT1 (n = 11 BGCs). We observed a weak but significant positive correlation between genome size and the number of BGCs per genome (R^2^ = 0.29458, p-value = 0.0) (Fig. 2b).

The most common BGCs were non-ribosomal peptide synthetases (NRPS; present in 1,062 genomes), type 1 polyketide synthases (t1PKS; 981 genomes), terpenes (697 genomes), other ketide synthases (KS; 650 genomes) and lantipeptides (540 genomes) (Fig. 2c). These five classes of BGCs comprised approximately half of the total BGCs found in any single genome. We also note that a strain may harbor multiple copies of a BGC class. For example, the maximum number of NRPS in a single genome was 60 (found in *Streptomyces rimosus* subsp. *rimosus*), while the average number of NRPS per genome is 17.66. Other BGCs that were commonly found in *Streptomyces* include butyrolactone (present in 503 genomes), Type 2 PKS (499 genomes), bacteriocin (419 genomes), and Type 3 PKS (366 genomes). Some BGCs were rarely found and were present in only a handful of genomes. These include bottromycin (17 genomes), furan (15 genomes), phosphoglycolipid (12 genomes), homoserine lactone (8 genomes), cyanobactin (7 genomes), acyl aminoacids (2 genomes), and resorcinol (1 genome).

Some BGCs contain genes that code for more than one type of scaffold-synthesizing enzymes and are called hybrid BGCs^20,21^. The origins and specific roles of these hybrid BGCs are not fully known, but they provide additional structural and chemical modifications in major classes of BGCs and may produce medically beneficial derivatives of a compound^22,23^. If we subdivide the 34 BGCs into their hybrid types, we obtain a total of 541 unique BGCs. The most common hybrid BGCs were Type 1 PKS-NRPS (present in 753 genomes), Type 1 PKS-other types of KS (237 genomes), terpene-NRPS (189 genomes), bacteriocin-lantipeptide (143 genomes), and Type 3 PKS-NRPS (131 genomes) (Fig. 2d). Of the 1,110 genomes, a total of 1,088 genomes, representing 98% of the dataset, carry at least one hybrid BGC (mean number of hybrid BGCs per genome = 5.58, s.d. = 2.92). *Streptomyces griseochromogenes* ATCC 14511 has the highest number of hybrid BGCs (n = 19). However, it must be kept in mind that the draft nature of many of these genomes is likely to affect BGC prediction by antiSMASH.

### BGCs with known chemotherapeutic potential are found in multiple species

Some *Streptomyces* species are known to harbor BGCs that encode for secondary metabolites with antitumor activity, which we refer to as chemotherapeutic gene clusters (CGCs) to differentiate them from other BGCs. Using individual genes of each CGC obtained from DoBISCUIT (Supplementary Table S2) as query sequences, we searched all *Streptomyces* genomes for the presence of 38 CGCs from DoBISCUIT using BLASTX^24^. We defined the presence of a CGC if at least 90% of the individual genes that comprise a CGC have significant BLASTX hits (minimum e-value of 10^−10^). These minimum threshold values were selected to maintain a conservative approach to detecting CGCs and account for possible genome sequencing errors. We also searched the NCBI database for the genome sequences of strains listed in DoBISCUIT. Of all the strains from the DoBISCUIT database that encode the 38 CGCs, only two strains (*Streptomyces globisporus* C-1027 for the compound C-1027 [synonym: lidamycin] and *Streptomyces neyagawaensis* ATCC 27449 for concamycin) have genome sequences available in NCBI and were included in our dataset. To ensure that our approach of CGC detection is accurate, we searched for the BGCs that encode for these compounds in the genomes of the two *Streptomyces* species. Results indicate that we were able to retrieve the CGCs for C-1027 and concamycin in these genomes, thereby ensuring the reliability of our output.

**Figure 3:**
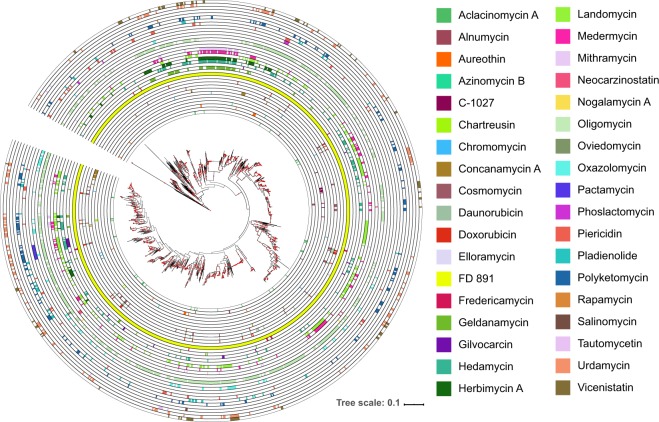
Phylogenetic distribution of the CGCs that encode antitumor compounds. Presence of each CGC was determined by searching all the genomes for homologs of each of the genes comprising the CGCs using BLASTX^24^ with a minimum e-value of 10^−10^. Sequences of individual genes in a CGC were obtained from DoBISCUIT^57^ and were used as query sequences. Presence of the CGC was inferred if there were significant BLASTX hits for at least 90% of the individual genes within the CGC. The phylogenetic tree is identical to that used in Fig. 1.

We found that CGCs are widely but differentially distributed in the *Streptomyces* phylogeny (Fig. 3 and Supplementary Table S3). The most common CGCs were FD-891 and oligomycin, which were present in 1,109 and 606 genomes, respectively. The macrolide FD-891 was initially isolated from *Streptomyces graminofaciens* A-8890 and has been shown to have strong cytocidal activities against human promyelocytic leukemia (HL‐60) and Jurkat cells through its ability to induce apoptosis^25^. Oligomycin is another macrolide whose antitumor capabilities originate from the inhibition of the F_0_ site of ATP synthase, blocking proton conduction and ultimately inducing apoptosis^26^.

Induction chemotherapy based on anthracyclines remains a part of standard of care therapy for acute myeloid leukemia (AML) patients^27,28^. One of the most commonly used anthracycline for AML chemotherapy is daunorubicin^29,30^. It was first isolated from *Streptomyces* sp. C5 and acts mainly through intercalation with DNA and inhibition of macromolecular biosynthesis^31^. We found 40 *Streptomyces* genomes that carry the BGC encoding for daunorubicin (Fig. 3). Other anthracyclines, such as aclacinomycin A from *Streptomyces galilaeus* ATCC 31615, doxorubicin from *Streptomyces peucetius* ATCC 29050 (=NBRC 100596), and nogalamycin from *Streptomyces nogalater* ATCC 27451 (=NBRC 13445) were also found in 37, 32, and 2 genomes, respectively.

Of the 38 antitumor CGCs from DoBISCUIT, we did not find steffimycin (anthracycline) and tautomycin (tetronic acid derivative) in any of the genomes in our dataset. However, we hypothesized that these genomes are likely to carry some of the genes of these three CGCs but may not have the minimum 90% of the genes of a CGC, which we used as a cut-off to define the presence of a CGC (Supplementary Fig. S1). We found that all *Streptomyces* genomes harbor one or few of the genes of steffimycin and tautomycin CGCs. We also found that sequence similarity and patterns of presence/absence vary across the individual genes in each of the 38 CGCs (Supplementary Table S3). Overall, we found numerous genomes that carry diverse classes of CGCs, but because our cluster completeness and e-value threshold values err on the conservative side, we predict that the number of CGCs may be significantly higher than what were identified using our threshold values.

### Strain-level variation in BGC distribution

**Figure 4:**
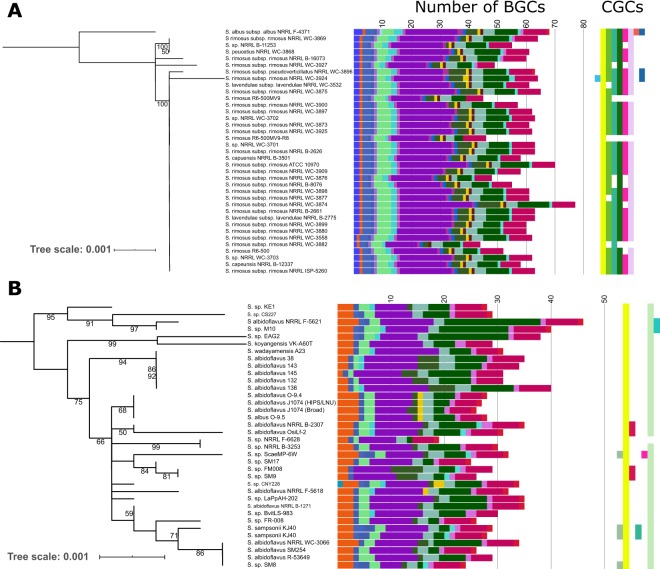
Inter-strain differences in the distribution of BGCs and CGCs. Two sub-clusters with nearly identical *rpoB* sequences were obtained from the phylogeny in Fig. 1 (branches labeled orange [**A**] and blue [**B**]). Colors of BGCs are identical to those in Figs. 1 and 3. Scale bars of both midpoint-rooted phylogenetic trees represent nucleotide substitutions per site. For visual clarity, only bootstrap values ≥ 50% are shown.

BGC analyses in Actinobacteria are often done using a single strain representative of a species. A few recent studies, however, have highlighted major differences in biosynthetic diversity even among very closely related strains^32,33^, which may represent an attractive yet untapped reservoir of novel compounds. Here, we explored the extent to which members of the same species differ in the abundance and diversity of BGCs they carry. We selected two sub-clusters in the *rpoB* phylogeny that have identical or near identical *rpoB* sequences (labeled in orange and blue branches on the tree in Fig. 1). While strains in each cluster are likely to represent the same species, the long history of misclassification in *Streptomyces* taxonomy^34^ has created conflicting species groupings. Nevertheless, we observed highly variable BGC composition among members of each phylogenetic cluster. In the orange cluster, with majority of strains related to *Streptomyces rimosus*, each genome carried between 44–77 BGCs from the 34 major classes of BGCs (Fig. 4a). We found the same pattern in the second cluster where majority of strains are named as *Streptomyces albidoflavus*, and genomes harbored between 19–46 BGCs from the 34 major BGC classes (Fig. 4b). We also found variation in the distribution of CGCs among closely related strains. Overall, strain-level comparison of BGC diversity revealed that individual members of the same species vary in their BGC composition.

## Discussion

In this study, we aimed to explore the phylogenetic distribution and diversity of BGCs in 1,110 *Streptomyces* genomes. We showed that BGCs and CGCs in *Streptomyces* are highly diverse and exhibit variable patterns of phylogenetic distribution, with hybrid BGCs and inter-strain variation further expanding the BGC repertoire present in any one genome. These findings are consistent with recent reports in other genera of Actinobacteria^6,18,19^ and also highlight the power of mining the genomes of closely related strains. In a recent study of ten *Streptomyces* strains with 16S rRNA gene sequences that are all identical to the type strain of *Streptomyces cyaneofuscatus* and hence are considered the same species, no two strains appear to exhibit identical secondary metabolomic profiles^32^. In another example, *Streptomyces albus*, a remarkable 48 BGCs were identified in a mere seven strains^33^. Of these, 18 are found in all strains and can be considered as the core BGCs of the species, 14 are ancillary or accessory BGCs (present in some strains but not all) and 16 are unique to an individual strain^33^.

The highly variable phylogenetic distribution of BGCs, even among closely related *Streptomyces* genomes, is indicative of recent gene loss and horizontal gene transfer (HGT) events^35,36^, which is prevalent in *Streptomyces*^35–38^. Furthermore, the existence of hybrid BGCs, composed of different scaffold-synthesizing enzymes from the major BGC classes and joined in a variety of combinations^22,23^, further reinforce the role of these two processes in generating the remarkable BGC diversity in *Streptomyces*. That BGCs have undergone frequent loss and HGT is not surprising. Genes that encode phenotypes that are subjected to weak selection are likely to be lost, but can be regained via HGT when they confer immediate positive benefit in certain conditions^39^. Hence, inter-strain BGC variation should be taken into consideration when developing approaches to discover microbe-derived pharmacological compounds and that reliance on a single gene (e.g., 16S rRNA, *rpoB*) is likely to result in sampling strategies that overlook possible novel compounds with drug potential. Moreover, our conservative threshold values (90% cluster completeness and e-value of 10^−10^) are likely to overlook the presence of BGCs in majority of the *Streptomyces* genomes and our results may only be the tip of the BGC iceberg found in this genus. Future work should therefore emphasize deep sequencing methods (i.e., sequencing a genomic region hundreds or even thousands of times) to precisely clarify the presence and gene content characteristics of specific BGCs in a genome.

One possible explanation to account for the existence of the differential distribution of BGCs between strains is that it may prevent the complete loss of a BGC from the population. Even a rare BGC can potentially be beneficial to the entire population and subsequently becomes more common via frequent HGT between strains. As has been observed in the genus *Salinispora*, the different ways by which BGCs are re-assorted between strains to produce hybrid BGCs suggest the major role of HGT in the production of structurally and/or functionally unique secondary metabolites^18^. This likely holds true for *Streptomyces* as well and may likely be medically relevant. A previous study reported the production of multiple antibiotics by symbiotic *Streptomyces* harbored by beewolf digger wasps that the insect uses to protect their larvae and cocoons^40^. Comparable to the combination antimicrobial prophylaxis commonly used to treat human patients, this strategy carried out by the bacterial population, and not simply by individual strains, makes use of the synergistic action of eight different compounds, thereby providing an important long-term and more efficacious defense against multiple diseases^40^. Hence, personalized medical strategies in the future can be developed towards using specific combinations of *Streptomyces* bacteria to produce drugs that will target multiple medical conditions. However, this concept remains a hypothesis and the extent in which bacteria make use of a population-level BGC variation clearly requires a more in-depth investigation and sequencing a greater number of closely related, co-existing strains.

The observation that majority of the *Streptomyces* genomes carry some of the individual genes in a CGC while only a few harbor the nearly complete set of genes, may be explained by a cooperative strategy conceptualized in the Black Queen hypothesis^41,42^. Here, accessory genes, including BGCs, can be viewed as a shared resource in which members of a microbial population or community make use of the compounds or functions derived from close relatives or other taxa^41,42^. Hence, mutual sharing of common goods will lead to mutual dependencies and cooperation among members of a group^41,42^. On the other hand, the differential distribution of individual genes of the 38 CGCs in *Streptomyces* suggests that there exists a common set of genes and pathways in the production of each CGC^43^. The differences in the distribution of other components of the CGCs may suggest that each species or strain may produce different derivatives of each of the 38 chemotherapeutic natural products^43^, which may prove useful in discovering more effective drugs for a variety of diseases. However, we acknowledge that this is a hypothesis and future work should therefore focus on elucidating the dynamics of ecological interactions between strains in a population.

Our study presents several caveats. A major caveat of this study is that current methods of BGC identification, including methods used in this study, are largely dependent on the composition of the BGC database being used for comparison. This means that BGCs that encode for metabolites with previously unrecognized functions or cellular targets may be missed. Moreover, the draft nature of many of the genomes can have a major bearing on the ability of antiSMASH to accurately predict BGCs, particularly so in correctly identifying hybrid BGCs. Hence, improved sequencing quality is likely to alter some of our results. Second, our analyses included only the major classes identified by antiSMASH, which represents the broad diversity of BGC secondary metabolite products. Future work should therefore emphasize the extent of BGC diversity at a more fine-scale resolution (e.g., structural subclasses of each BGC). Another weakness of the study is the use of a single-gene (*rpoB*) phylogenetic tree. While *rpoB* has been used in initial identification and classification of *Streptomyces* in previous work^44–46^, future *Streptomyces* studies will certainly benefit from using genome-based data (e.g., average nucleotide identity [ANI]^47^) to clarify evolutionary relationships within and between species. We also point out that while this work does not aim to detect novel antibiotic compounds and other secondary metabolites, it provides important insights into the tremendous biosynthetic potential of the genus *Streptomyces* even below the species level. Future studies on inter-strain genomic variation as well as the ecological and evolutionary processes that shape it will have broad and positive impact on current efforts to explore the biosynthetic potential that exists in nature.

## Conclusions

There are two main conclusions from this study. First, we found high diversity and abundance of BGCs across the genus *Streptomyces*, with hybrid BGCs greatly expanding the repertoire of secondary metabolites and can therefore facilitate the discovery of novel pharmaceuticals. We also found that numerous *Streptomyces* species harbor BGCs known to encode antitumor compounds, and hence, represent important but generally untapped sources of chemotherapeutic agents. Second, we also observed that members of the same species can vary tremendously in the BGCs they carry, suggesting that strain-level genome sequencing can uncover high levels of BGC diversity and potentially useful derivatives of any one compound. These findings suggest that within-species sequencing strategy for exploring secondary metabolites for clinical use, instead of focusing on individual strains representative of a species, can provide an alternative or complementary approach to discovering novel compounds from microbes.

## Materials and Methods

### *Streptomyces* dataset

A total of 1,157 genomes of *Streptomyces* (as of September 2018) were downloaded from the National Center for Biotechnology Information (NCBI). Accession numbers and genomic information (genome size, % GC content, number of genes, number of protein-coding genes) are shown in Supplementary Table S1. Different annotation procedures and annotation assessment criteria can potentially introduce misannotations, missing genetic features and out-of-date information, which can remain unchecked and errors can then be propagated in future studies^48,49^. To ensure the utilization of up-to-date and discover potentially novel annotations for BGC prediction as well as maintain consistency in gene annotations, we re-annotated the *Streptomyces* genomes using Prokka, a pipeline comprising several programs that include locating open reading frames (ORFs) and RNA regions on contigs, translating ORFs to protein sequences, searching for protein homologs and producing standard output files for downstream applications^50^. A total of 47 genomes were excluded from downstream analyses due to poor quality of assemblies, highly divergent *rpoB* sequences, and failed antiSMASH analyses^17^.

### Phylogenetic tree reconstruction

We extracted the *rpoB* sequences from the genome assemblies and aligned them using MAFFT^51^. Out of the 1,110 genomes, three were missing the *rpoB* gene and were not therefore included in the phylogenetic tree reconstruction. The *rpoB* sequences were used to build a maximum likelihood phylogeny using the program RAxML v.8.2.11^52^ with a general time reversible (GTR) nucleotide substitution model^53^, four gamma categories for rate heterogeneity, and 100 bootstrap replicates (Supplementary Datasets S4 and S5). We used the *rpoB* locus instead of the 16S ribosomal RNA (rRNA) for two reasons. First, *Streptomyces* are known to harbor multiple copies of the rRNA operon, with as many as six copies and some of which are divergent^54,55^. Second, sequence variation in *rpoB* permits species differentiation and has been widely used for initial taxonomic identification of multiple *Streptomyces* species in previous studies^44–46^. We also ran RAxML on each of the two subclusters in Fig. 1 (labeled blue and orange) and were midpoint rooted. Phylogenetic trees were visualized using the Interactive Tree of Life [iToL]^56^.

### Identification of BGCs

BGCs encoding secondary metabolites were predicted and annotated using the standalone version of antiSMASH 4.1, which identifies BGCs using a signature profile Hidden Markov Model based on multiple sequence alignments of experimentally characterized signature proteins or protein domains^17^. Sequences of BGCs known to encode natural products with antitumor properties were obtained from DoBISCUIT (Database of BIoSynthesis cluster CUrated and InTegrated)^57^ (downloaded in March, 2019). We refer to these BGCs as chemotherapeutic gene clusters (CGCs) to differentiate them from BGCs that encode non-antitumor compounds. A total of 47 CGCs were listed in DoBISCUIT. However, we restricted our analyses to CGCs that were first isolated from *Streptomyces* bacteria. The compounds geldanamycin and salinomycin were listed twice in DoBISCUIT and each was reported to have been derived from two different *Streptomyces* strains. For clarity, we only used one copy of geldanamycin and salinomycin BGCs. At the end, we used a total of 38 CGCs in our analysis. We searched for the presence of these 38 CGCs representing 17 major classes of antitumor drugs (Supplementary Table S2) in 1,110 *Streptomyces* genomes using BLASTX and a minimum e-value of 10^−10^. Individual genes in a CGC were used as query sequences (Supplementary Table S3).

## Supplementary information

Supplementary Figure S1.

Supplementary Table S1.

Supplementary Table S2.

Supplementary Table S3.

Supplementary Table S4.

Supplementary Table S5.

## Data Availability

The datasets analyzed in this study were downloaded from and are available in the GenBank database (https://www.ncbi.nlm.nih.gov/genbank/). Accession numbers are listed in Supplementary Table S1.
