# Supplementary material for: Genome mining of biosynthetic and chemotherapeutic gene clusters in Streptomyces bacteria
Source: Sci Rep. 2020 Feb 6;10:2003. doi: 10.1038/s41598-020-58904-9 (PMC7005152; doi:10.1038/s41598-020-58904-9)
Supplement: Supplementary file 1 — Supplementary Figure S1. [file 41598_2020_58904_MOESM1_ESM.pdf]

Genome mining of biosynthetic and chemotherapeutic gene clusters in *Streptomyces* bacteria

Supplementary information

Kaitlyn C. Belknap, Cooper J. Park, Brian M. Barth and Cheryl P. Andam

University of New Hampshire, Department of Molecular, Cellular and Biomedical Sciences,

Durham, NH 03824, USA

Corresponding Author: Cheryl.Andam@unh.edu

Supplementary information

Supplementary Table S1. Accession numbers of the 1,110 *Streptomyces* genomes used in this study.

Supplementary Table S2. List of CGCs used in Figure 3. Sequences were obtained from DoBISCUIT.

Supplementary Table S3. List of individual genes per CGC and BLASTX results for individual genes in each CGC. Numbers represent the percentage sequence similarity to the query sequence.

Supplementary Dataset S4. Sequence alignment of *rpoB*

Supplementary Dataset S5. *rpoB* phylogenetic tree with bootstrap values

Supplementary Figure S1. Number of CGCs identified using different threshold values of cluster completeness and e-values. Numbers in blue squares are those that were included in the study.

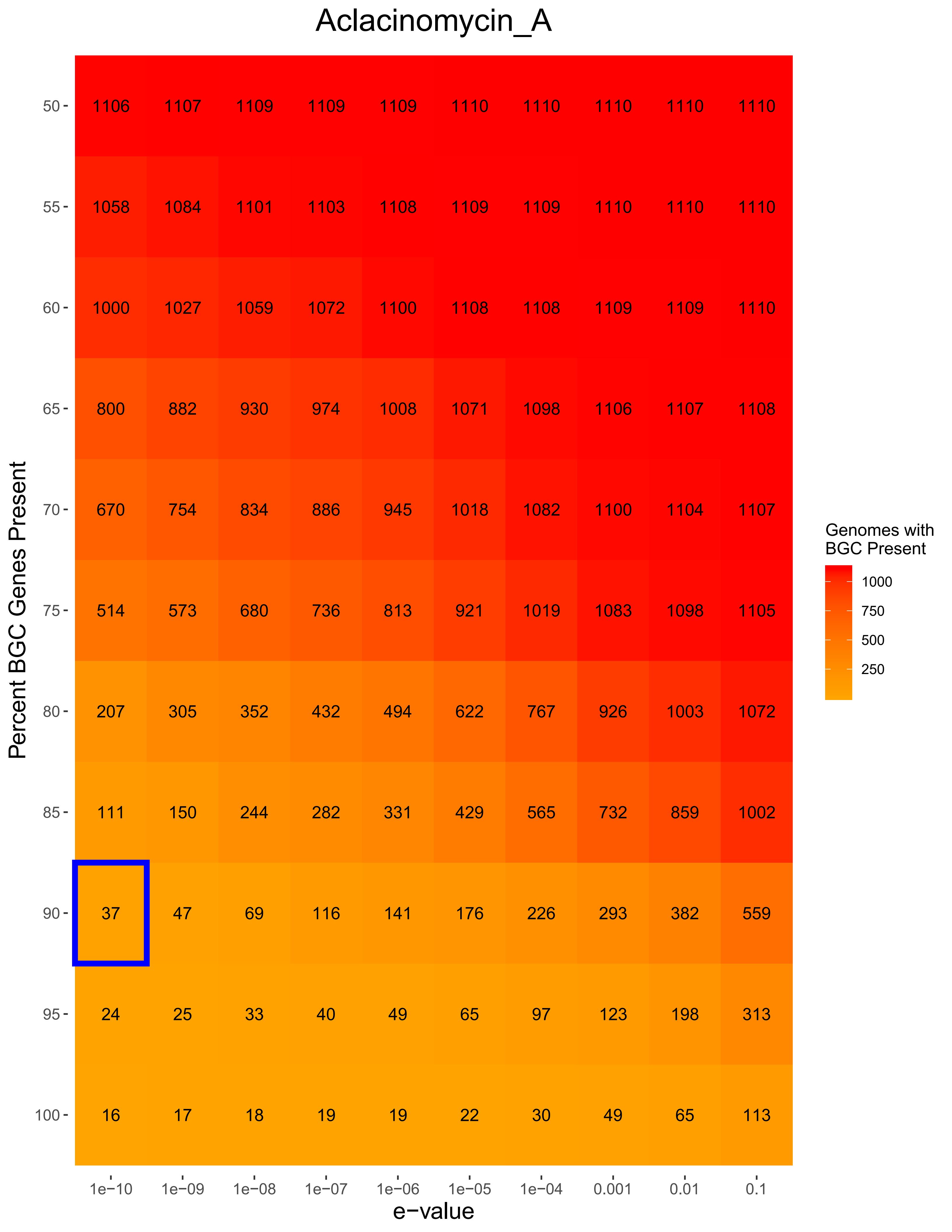

Aclacinomycin_A
Percent BGC Genes Present
e−value
1e−10
1e−09
1e−08
1e−07
1e−06
1e−05
1e−04
0.001
0.01
0.1
50
1106 1107 1109 1109 1109 1110 1110 1110 1110 1110
55
1058 1084 1101 1103 1108 1109 1109 1110 1110 1110
60
1000 1027 1059 1072 1100 1108 1108 1109 1109 1110
65
800 882 930 974 1008 1071 1098 1106 1107 1108
70
670 754 834 886 945 1018 1082 1100 1104 1107
75
514 573 680 736 813 921 1019 1083 1098 1105
80
207 305 352 432 494 622 767 926 1003 1072
85
111 150 244 282 331 429 565 732 859 1002
90
37 47 69 116 141 176 226 293 382 559
95
24 25 33 40 49 65 97 123 198 313
100
16 17 18 19 19 22 30 49 65 113
Genomes with BGC Present
1000
750
500
250

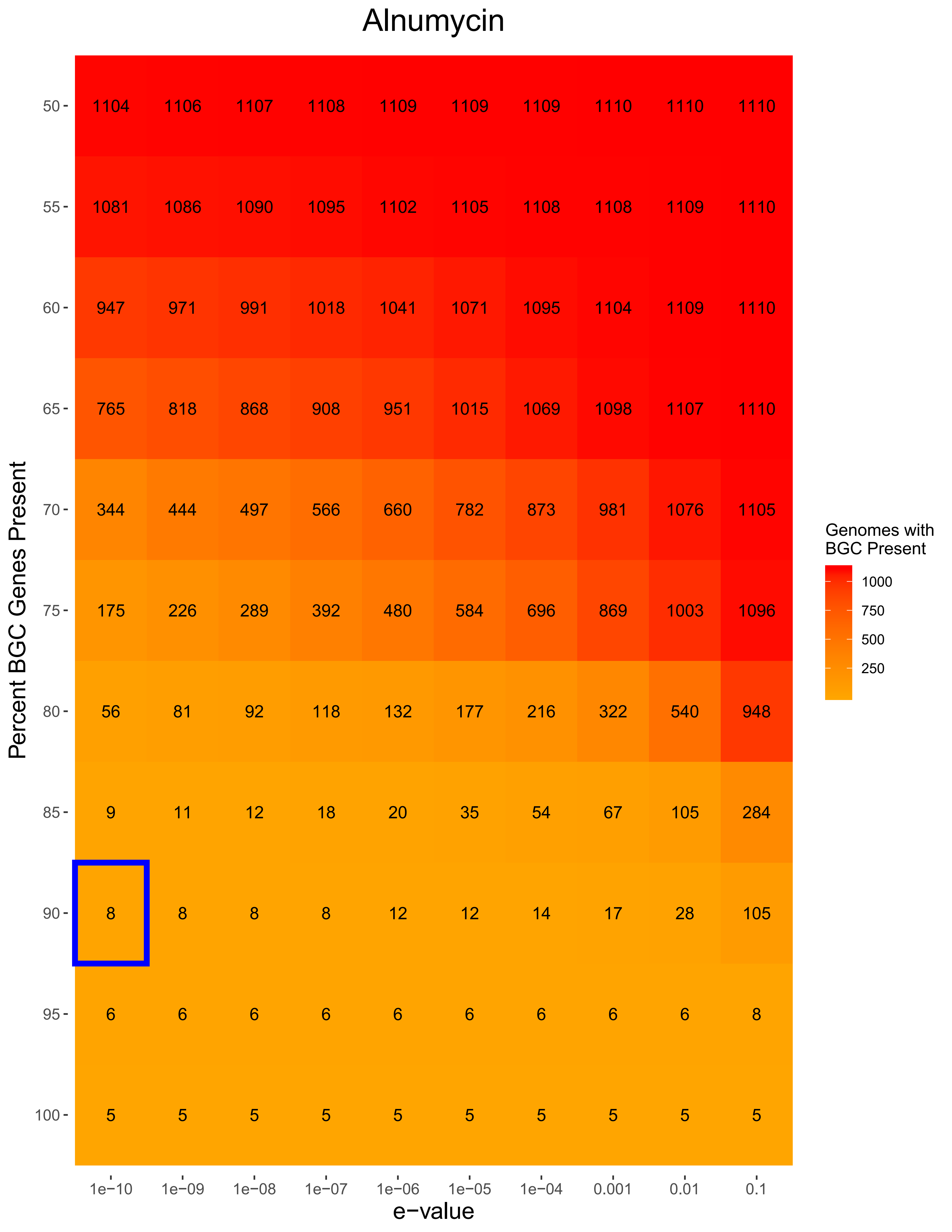
Alnumycin
Percent BGC Genes Present
e−value
Genomes with BGC Present
1000
750
500
250
50 55 60 65 70 75 80 85 90 95 100
1e−10 1e−09 1e−08 1e−07 1e−06 1e−05 1e−04 0.001 0.01 0.1
1104 1106 1107 1108 1109 1109 1109 1110 1110 1110
1081 1086 1090 1095 1102 1105 1108 1108 1109 1110
947 971 991 1018 1041 1071 1095 1104 1109 1110
765 818 868 908 951 1015 1069 1098 1107 1110
344 444 497 566 660 782 873 981 1076 1105
175 226 289 392 480 584 696 869 1003 1096
56 81 92 118 132 177 216 322 540 948
9 11 12 18 20 35 54 67 105 284
8 8 8 8 12 12 14 17 28 105
6 6 6 6 6 6 6 6 6 8
5 5 5 5 5 5 5 5 5 5

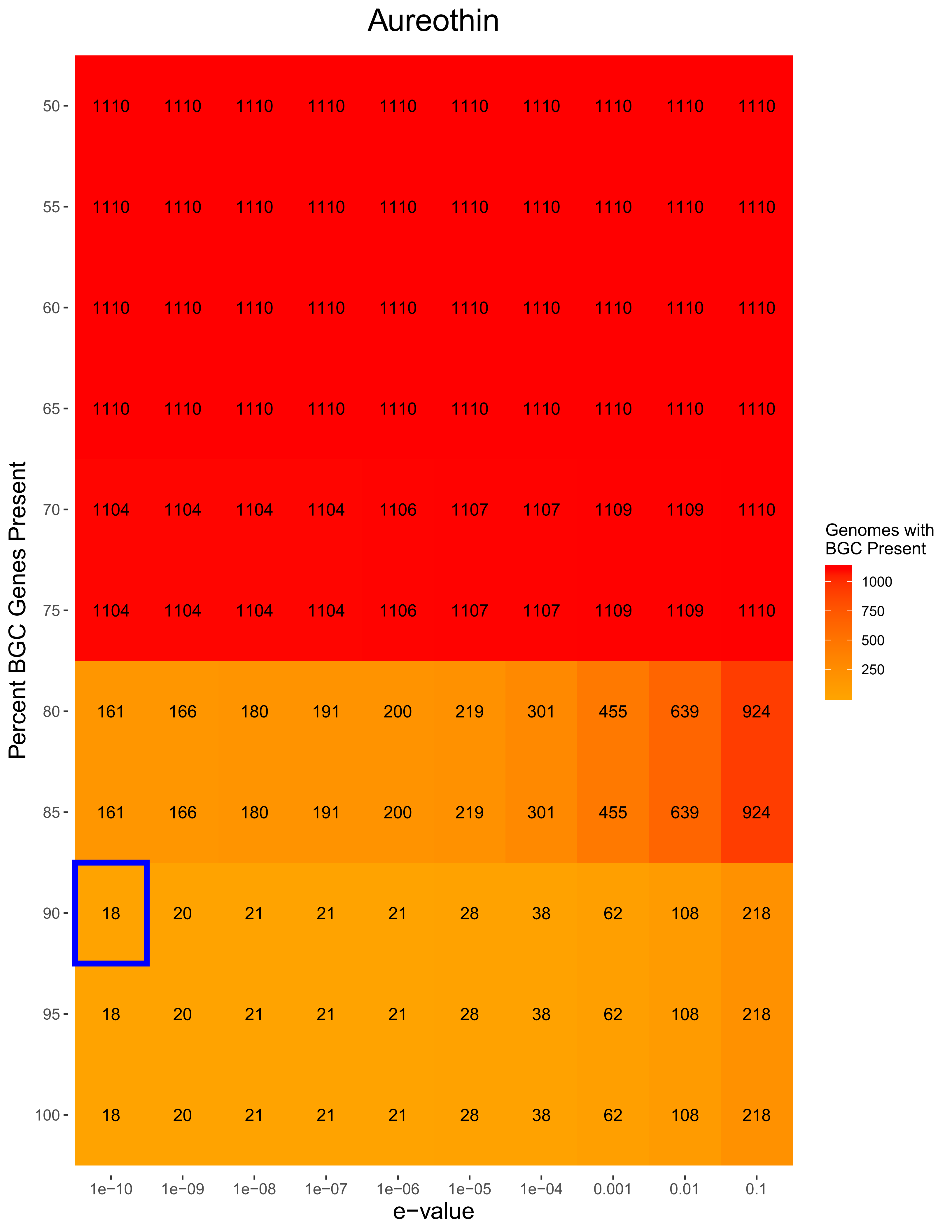

Aureothin
Percent BGC Genes Present
e−value
Genomes with BGC Present
1000
750
500
250
50
55
60
65
70
75
80
85
90
95
100
1e−10
1e−09
1e−08
1e−07
1e−06
1e−05
1e−04
0.001
0.01
0.1
1110 1110 1110 1110 1110 1110 1110 1110 1110 1110
1110 1110 1110 1110 1110 1110 1110 1110 1110 1110
1110 1110 1110 1110 1110 1110 1110 1110 1110 1110
1110 1110 1110 1110 1110 1110 1110 1110 1110 1110
1104 1104 1104 1104 1106 1107 1107 1109 1109 1110
1104 1104 1104 1104 1106 1107 1107 1109 1109 1110
161 166 180 191 200 219 301 455 639 924
161 166 180 191 200 219 301 455 639 924
18 20 21 21 21 28 38 62 108 218
18 20 21 21 21 28 38 62 108 218
18 20 21 21 21 28 38 62 108 218

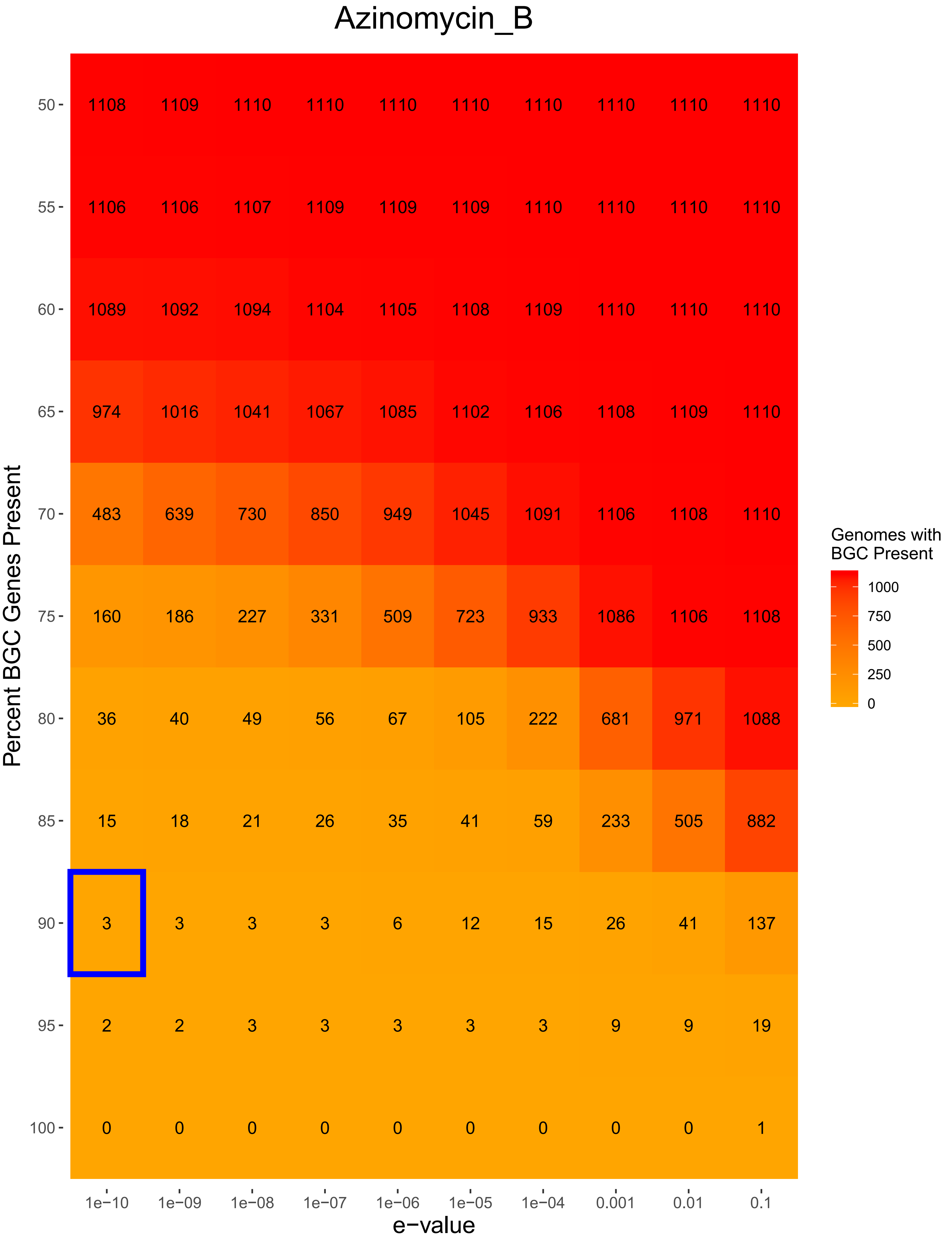
Azinomycin_B
Percent BGC Genes Present
e−value
Genomes with BGC Present
1000
750
500
250
0
1e−10 1e−09 1e−08 1e−07 1e−06 1e−05 1e−04 0.001 0.01 0.1
50 1108 1109 1110 1110 1110 1110 1110 1110 1110 1110
55 1106 1106 1107 1109 1109 1109 1110 1110 1110 1110
60 1089 1092 1094 1104 1105 1108 1109 1110 1110 1110
65 974 1016 1041 1067 1085 1102 1106 1108 1109 1110
70 483 639 730 850 949 1045 1091 1106 1108 1110
75 160 186 227 331 509 723 933 1086 1106 1108
80 36 40 49 56 67 105 222 681 971 1088
85 15 18 21 26 35 41 59 233 505 882
90 3 3 3 3 6 12 15 26 41 137
95 2 2 3 3 3 3 3 9 9 19
100 0 0 0 0 0 0 0 0 0 1

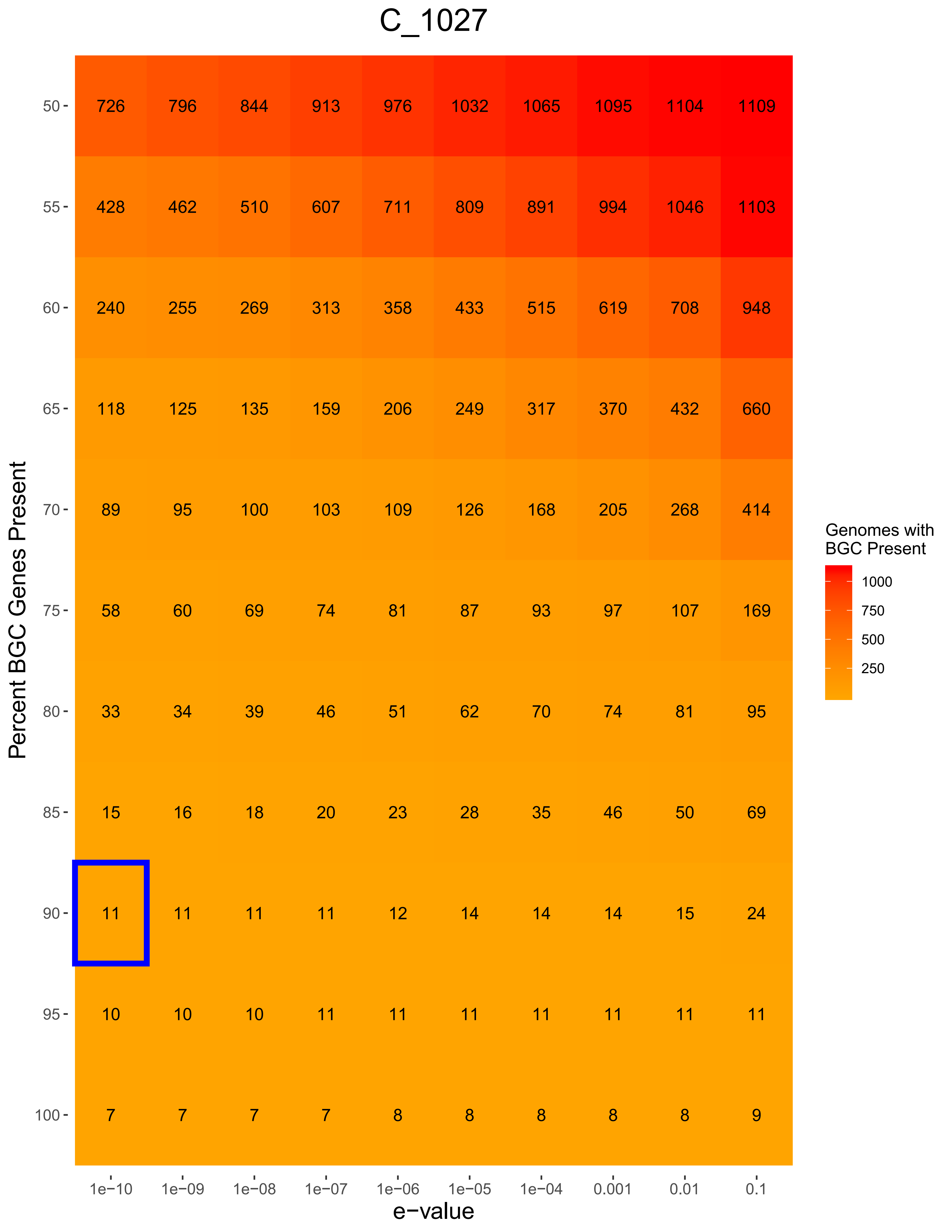
C_1027
Percent BGC Genes Present
e−value
Genomes with BGC Present
1000
750
500
250
50
55
60
65
70
75
80
85
90
95
100
1e−10
1e−09
1e−08
1e−07
1e−06
1e−05
1e−04
0.001
0.01
0.1
726 796 844 913 976 1032 1065 1095 1104 1109
428 462 510 607 711 809 891 994 1046 1103
240 255 269 313 358 433 515 619 708 948
118 125 135 159 206 249 317 370 432 660
89 95 100 103 109 126 168 205 268 414
58 60 69 74 81 87 93 97 107 169
33 34 39 46 51 62 70 74 81 95
15 16 18 20 23 28 35 46 50 69
11 11 11 11 12 14 14 14 15 24
10 10 10 11 11 11 11 11 11 11
7 7 7 7 8 8 8 8 8 9

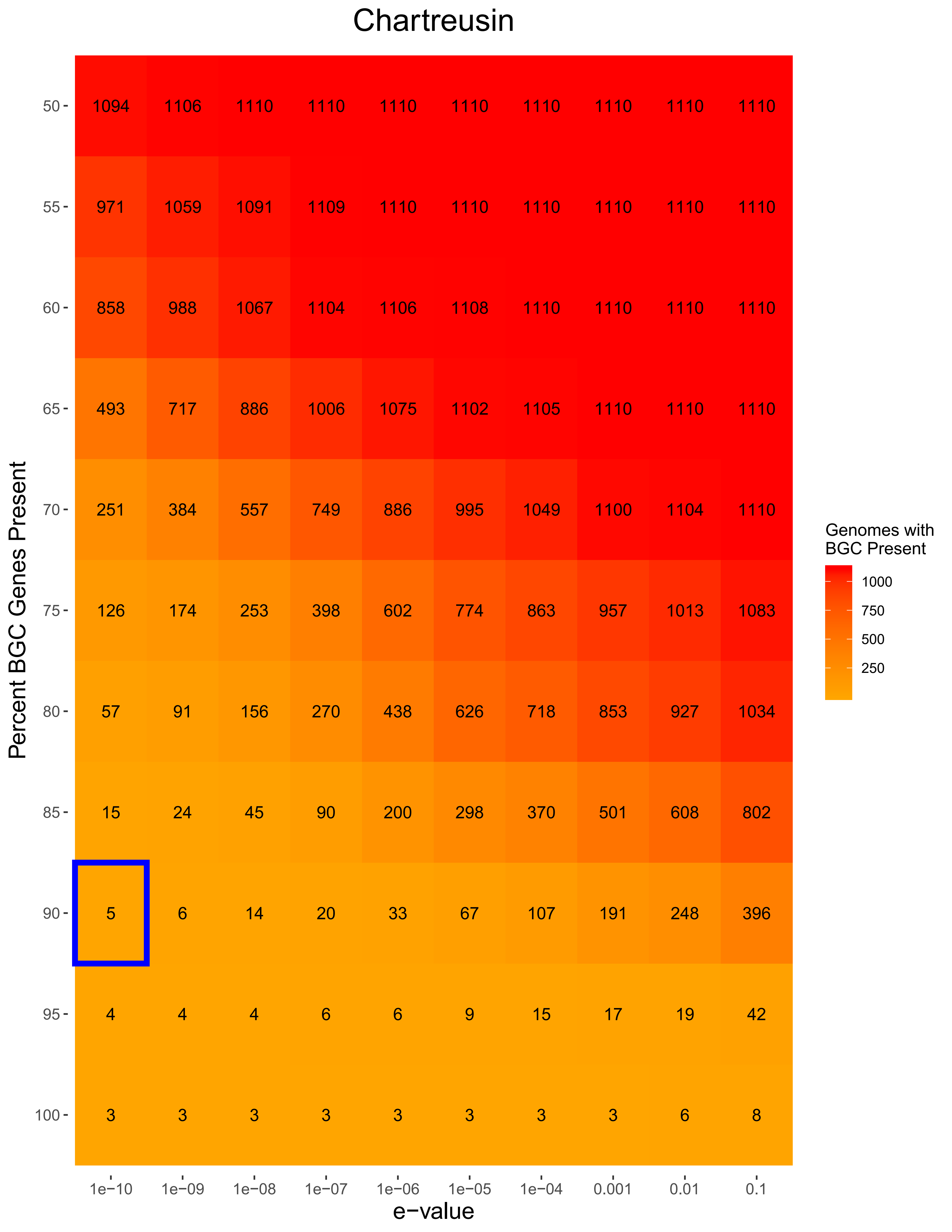
Chartreusin
Percent BGC Genes Present
e−value
Genomes with BGC Present
1000
750
500
250
50
55
60
65
70
75
80
85
90
95
100
1e−10
1e−09
1e−08
1e−07
1e−06
1e−05
1e−04
0.001
0.01
0.1
1094 1106 1110 1110 1110 1110 1110 1110 1110 1110
971 1059 1091 1109 1110 1110 1110 1110 1110 1110
858 988 1067 1104 1106 1108 1110 1110 1110 1110
493 717 886 1006 1075 1102 1105 1110 1110 1110
251 384 557 749 886 995 1049 1100 1104 1110
126 174 253 398 602 774 863 957 1013 1083
57 91 156 270 438 626 718 853 927 1034
15 24 45 90 200 298 370 501 608 802
5 6 14 20 33 67 107 191 248 396
4 4 4 6 6 9 15 17 19 42
3 3 3 3 3 3 3 3 6 8

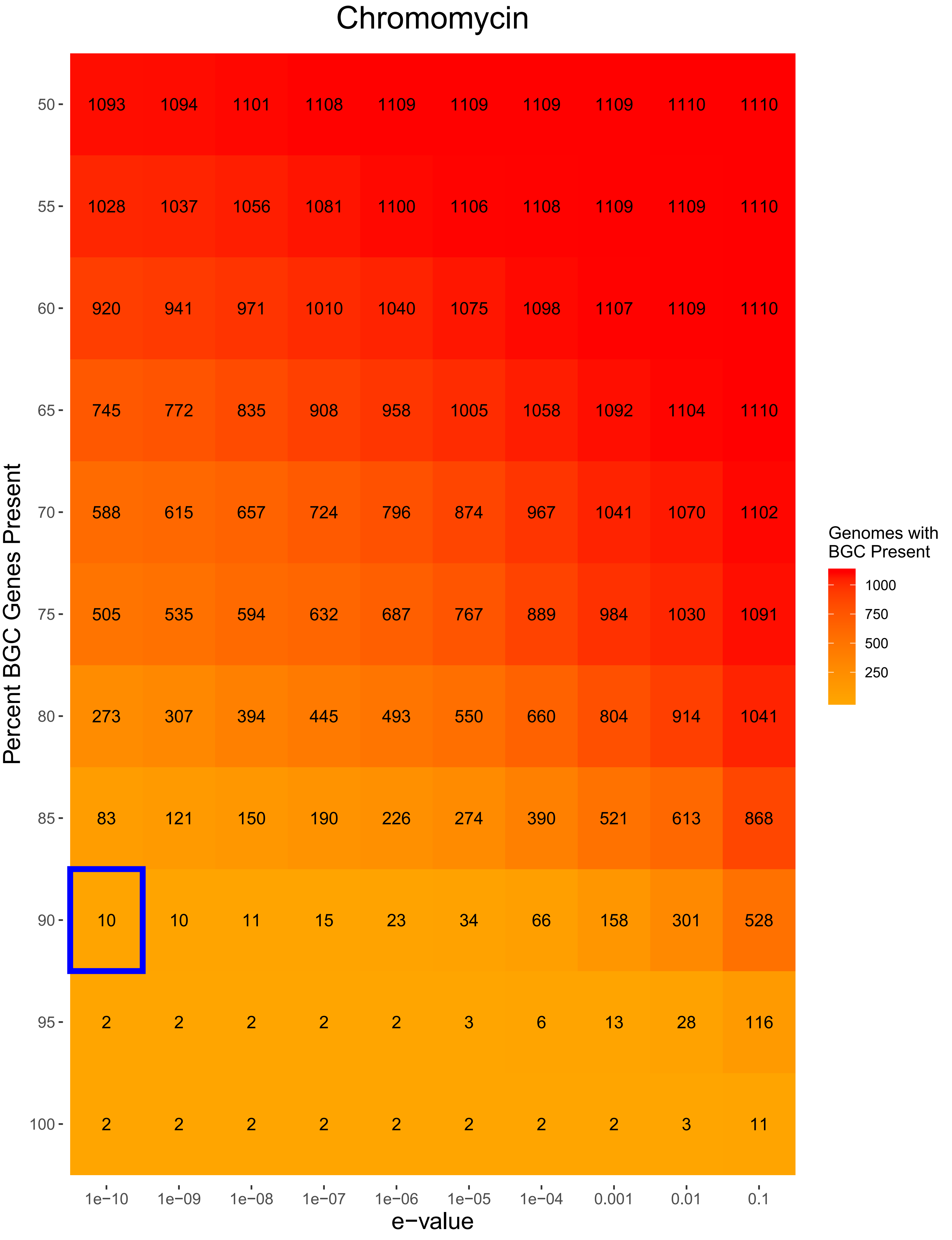
Chromomycin
Percent BGC Genes Present
e−value
1e−10
1e−09
1e−08
1e−07
1e−06
1e−05
1e−04
0.001
0.01
0.1
50
55
60
65
70
75
80
85
90
95
100
1093 1094 1101 1108 1109 1109 1109 1109 1110 1110
1028 1037 1056 1081 1100 1106 1108 1109 1109 1110
920 941 971 1010 1040 1075 1098 1107 1109 1110
745 772 835 908 958 1005 1058 1092 1104 1110
588 615 657 724 796 874 967 1041 1070 1102
505 535 594 632 687 767 889 984 1030 1091
273 307 394 445 493 550 660 804 914 1041
83 121 150 190 226 274 390 521 613 868
10 10 11 15 23 34 66 158 301 528
2 2 2 2 2 3 6 13 28 116
2 2 2 2 2 2 2 2 3 11
Genomes with
BGC Present
1000
750
500
250

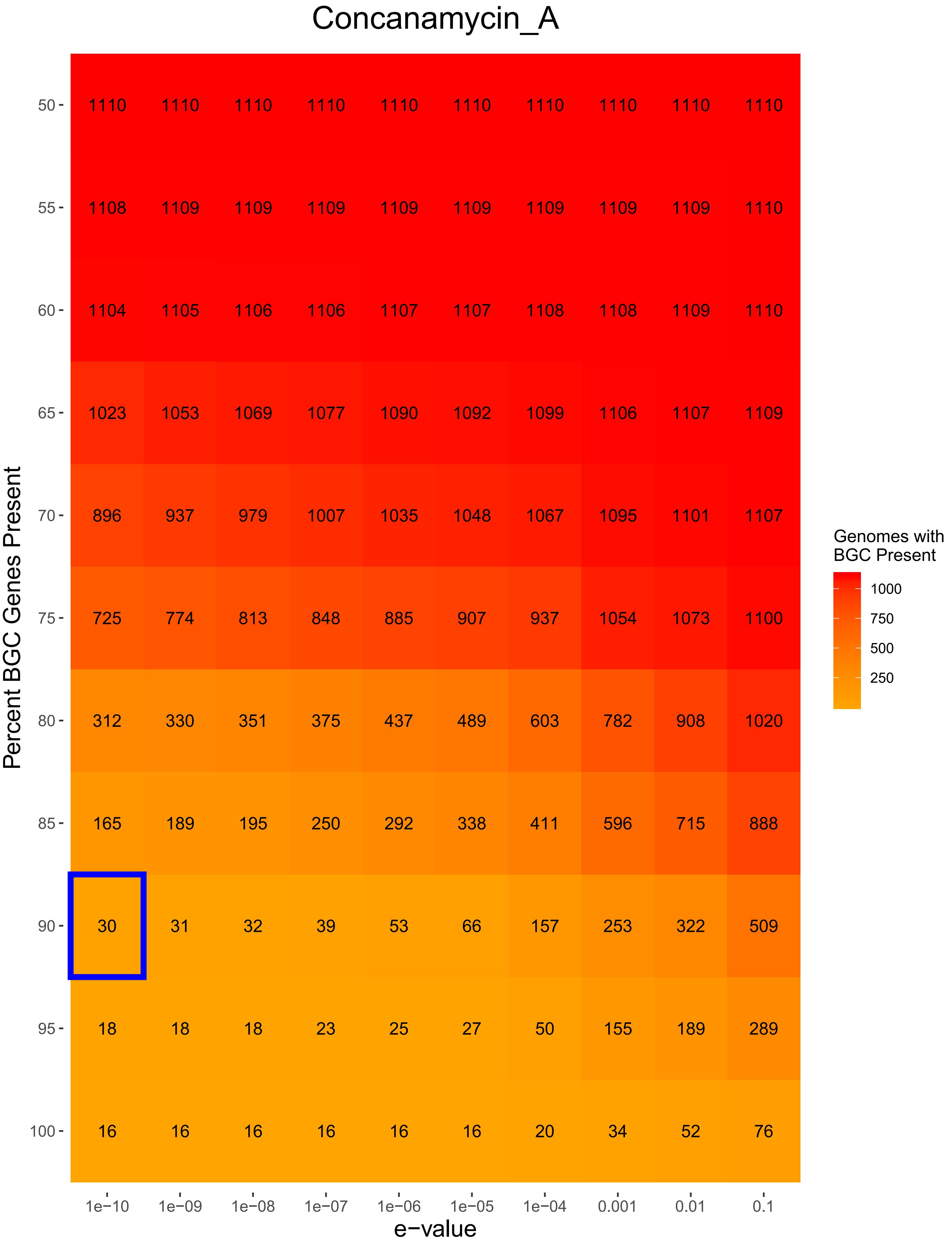

Concanamycin_A
Percent BGC Genes Present
e−value
Genomes with
BGC Present
1000
750
500
250
50
55
60
65
70
75
80
85
90
95
100
1e−10
1e−09
1e−08
1e−07
1e−06
1e−05
1e−04
0.001
0.01
0.1
1110 1110 1110 1110 1110 1110 1110 1110 1110 1110
1108 1109 1109 1109 1109 1109 1109 1109 1109 1110
1104 1105 1106 1106 1107 1107 1108 1108 1109 1110
1023 1053 1069 1077 1090 1092 1099 1106 1107 1109
896 937 979 1007 1035 1048 1067 1095 1101 1107
725 774 813 848 885 907 937 1054 1073 1100
312 330 351 375 437 489 603 782 908 1020
165 189 195 250 292 338 411 596 715 888
30 31 32 39 53 66 157 253 322 509
18 18 18 23 25 27 50 155 189 289
16 16 16 16 16 16 20 34 52 76

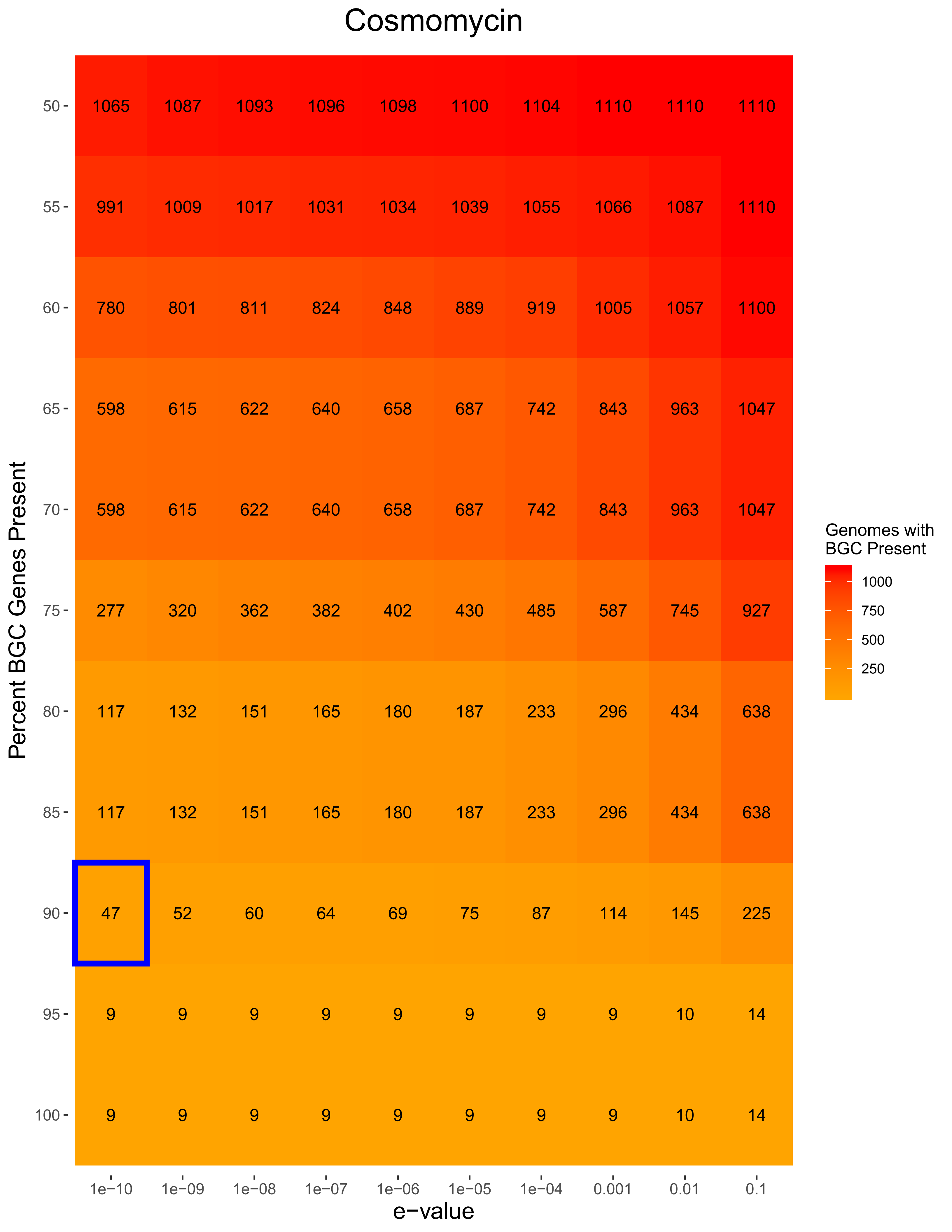

Cosmomycin
Percent BGC Genes Present
e−value
1e−10
1e−09
1e−08
1e−07
1e−06
1e−05
1e−04
0.001
0.01
0.1
50
55
60
65
70
75
80
85
90
95
100
1065 1087 1093 1096 1098 1100 1104 1110 1110 1110
991 1009 1017 1031 1034 1039 1055 1066 1087 1110
780 801 811 824 848 889 919 1005 1057 1100
598 615 622 640 658 687 742 843 963 1047
598 615 622 640 658 687 742 843 963 1047
277 320 362 382 402 430 485 587 745 927
117 132 151 165 180 187 233 296 434 638
117 132 151 165 180 187 233 296 434 638
47 52 60 64 69 75 87 114 145 225
9 9 9 9 9 9 9 9 10 14
9 9 9 9 9 9 9 9 10 14
Genomes with
BGC Present
1000
750
500
250

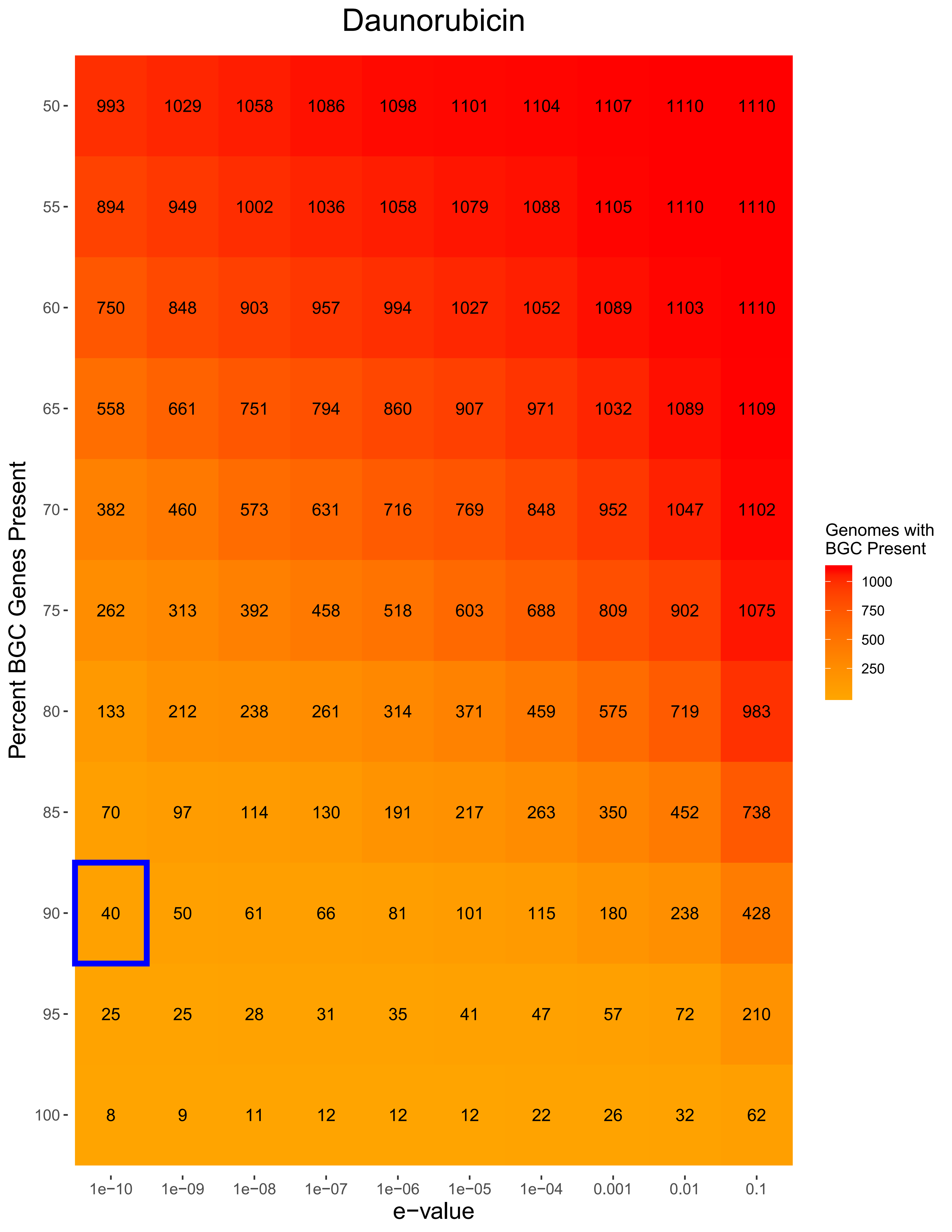
Daunorubicin
Percent BGC Genes Present
e−value
1e−10 1e−09 1e−08 1e−07 1e−06 1e−05 1e−04 0.001 0.01 0.1
50 993 1029 1058 1086 1098 1101 1104 1107 1110 1110
55 894 949 1002 1036 1058 1079 1088 1105 1110 1110
60 750 848 903 957 994 1027 1052 1089 1103 1110
65 558 661 751 794 860 907 971 1032 1089 1109
70 382 460 573 631 716 769 848 952 1047 1102
75 262 313 392 458 518 603 688 809 902 1075
80 133 212 238 261 314 371 459 575 719 983
85 70 97 114 130 191 217 263 350 452 738
90 40 50 61 66 81 101 115 180 238 428
95 25 25 28 31 35 41 47 57 72 210
100 8 9 11 12 12 12 22 26 32 62
Genomes with
BGC Present
1000
750
500
250

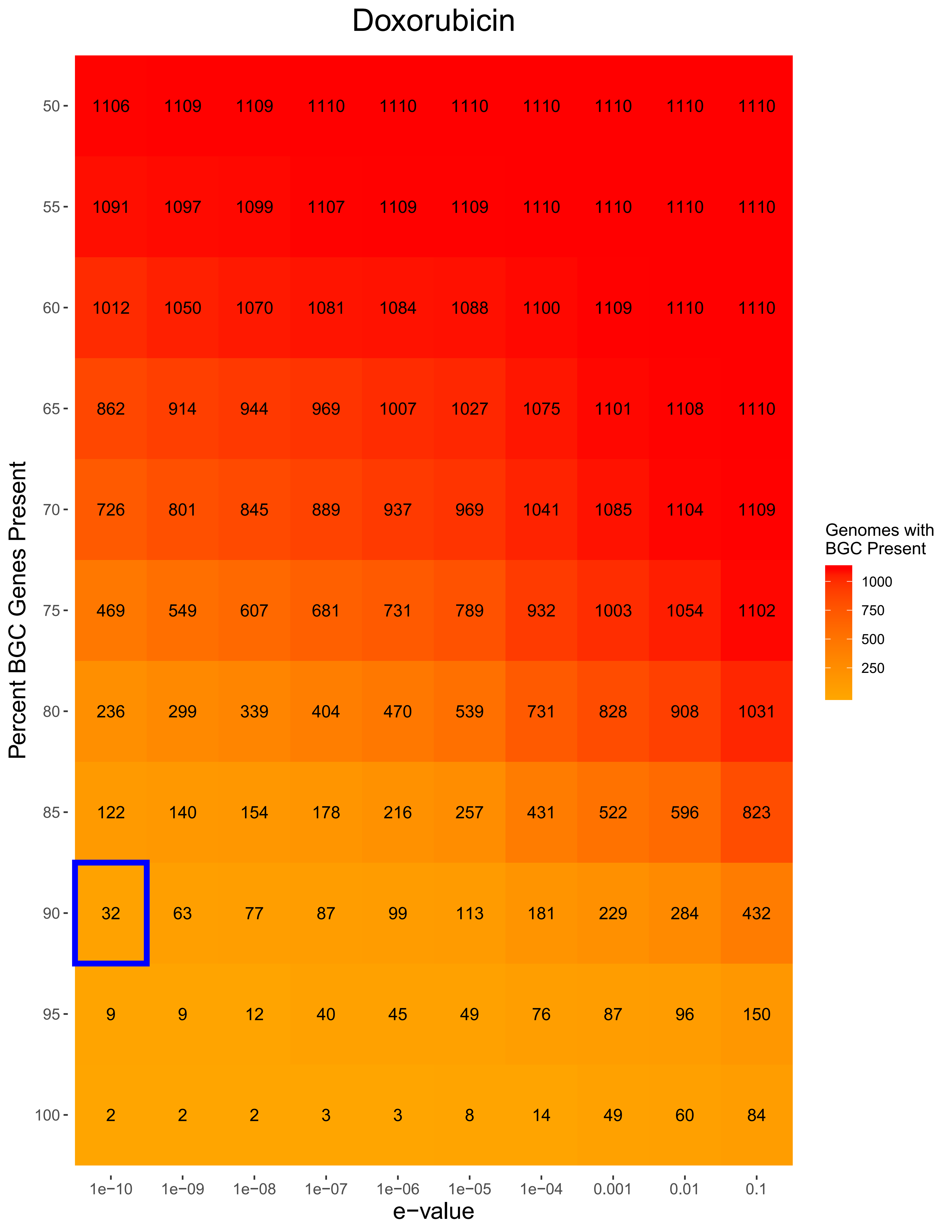

Doxorubicin
Percent BGC Genes Present
e−value
1e−10
1e−09
1e−08
1e−07
1e−06
1e−05
1e−04
0.001
0.01
0.1
50
55
60
65
70
75
80
85
90
95
100
Genomes with BGC Present
1000
750
500
250
1106 1109 1109 1110 1110 1110 1110 1110 1110 1110
1091 1097 1099 1107 1109 1109 1110 1110 1110 1110
1012 1050 1070 1081 1084 1088 1100 1109 1110 1110
862 914 944 969 1007 1027 1075 1101 1108 1110
726 801 845 889 937 969 1041 1085 1104 1109
469 549 607 681 731 789 932 1003 1054 1102
236 299 339 404 470 539 731 828 908 1031
122 140 154 178 216 257 431 522 596 823
32 63 77 87 99 113 181 229 284 432
9 9 12 40 45 49 76 87 96 150
2 2 2 3 3 8 14 49 60 84

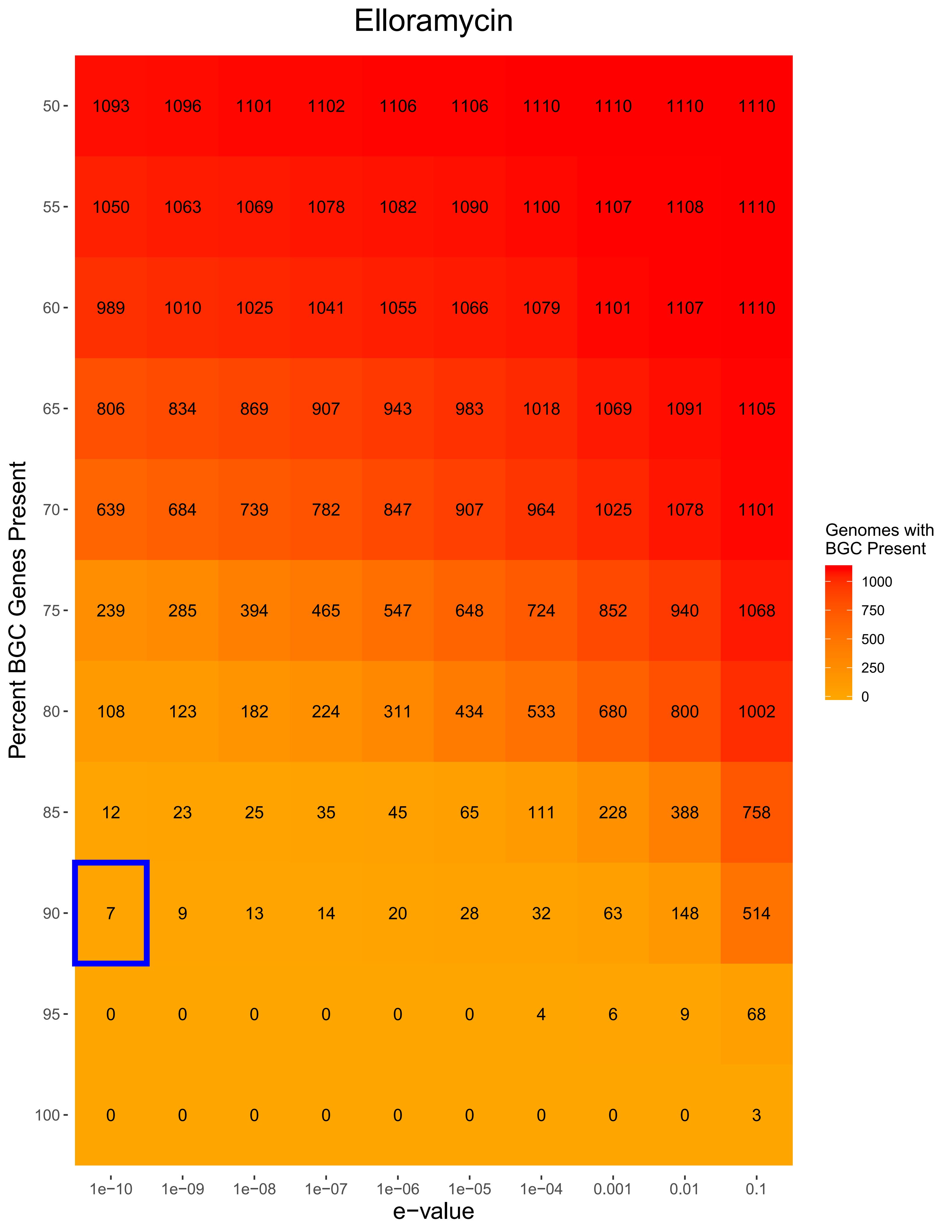
Elloramycin
Percent BGC Genes Present
e−value
1e−10 1e−09 1e−08 1e−07 1e−06 1e−05 1e−04 0.001 0.01 0.1
50: 1093 1096 1101 1102 1106 1106 1110 1110 1110 1110
55: 1050 1063 1069 1078 1082 1090 1100 1107 1108 1110
60: 989 1010 1025 1041 1055 1066 1079 1101 1107 1110
65: 806 834 869 907 943 983 1018 1069 1091 1105
70: 639 684 739 782 847 907 964 1025 1078 1101
75: 239 285 394 465 547 648 724 852 940 1068
80: 108 123 182 224 311 434 533 680 800 1002
85: 12 23 25 35 45 65 111 228 388 758
90: 7 9 13 14 20 28 32 63 148 514
95: 0 0 0 0 0 0 4 6 9 68
100: 0 0 0 0 0 0 0 0 0 3
Genomes with BGC Present
1000
750
500
250
0

FD_891

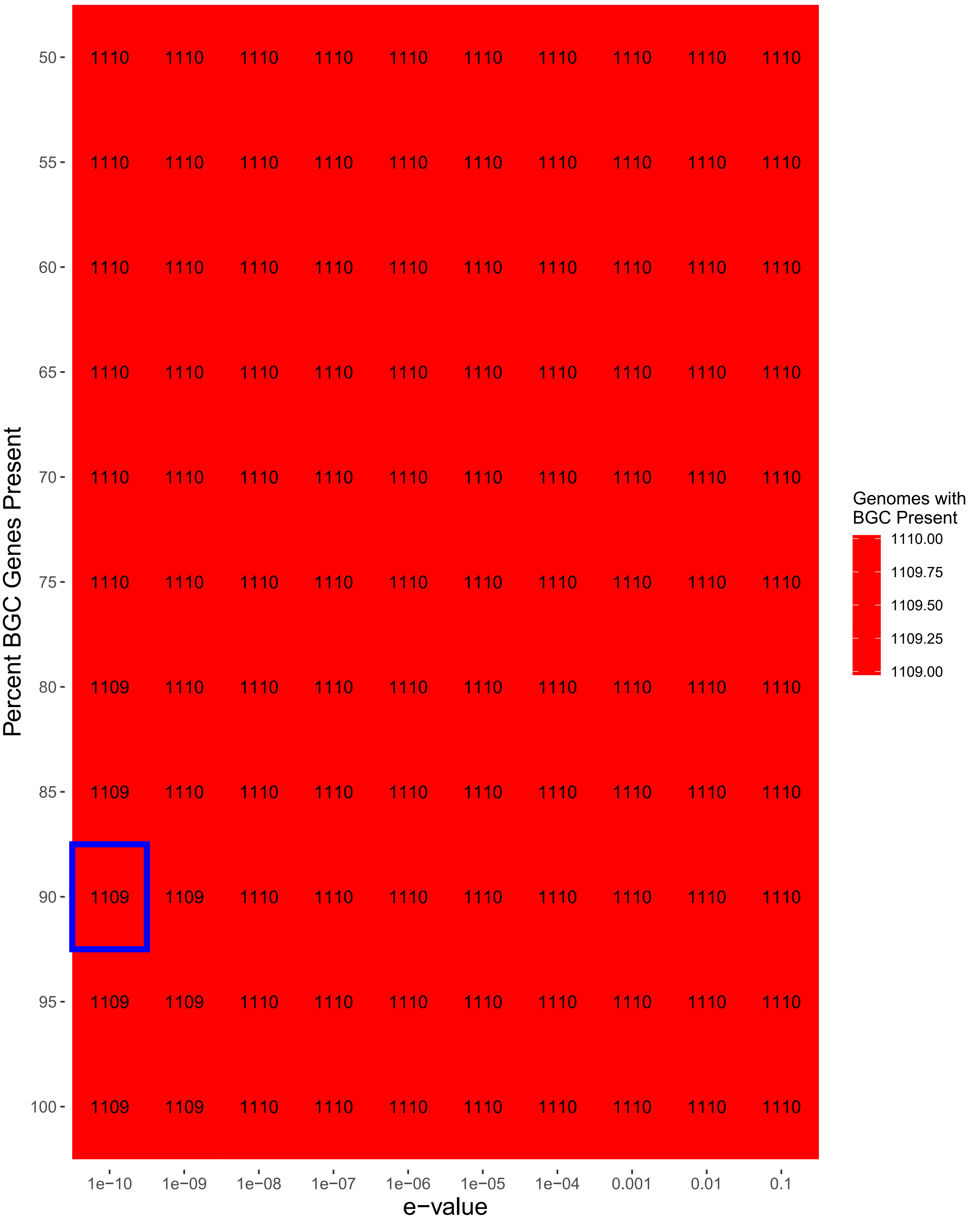
Percent BGC Genes Present
e−value
1e−10
1e−09
1e−08
1e−07
1e−06
1e−05
1e−04
0.001
0.01
0.1
50
55
60
65
70
75
80
85
90
95
100
1110 1110 1110 1110 1110 1110 1110 1110 1110 1110
1110 1110 1110 1110 1110 1110 1110 1110 1110 1110
1110 1110 1110 1110 1110 1110 1110 1110 1110 1110
1110 1110 1110 1110 1110 1110 1110 1110 1110 1110
1110 1110 1110 1110 1110 1110 1110 1110 1110 1110
1110 1110 1110 1110 1110 1110 1110 1110 1110 1110
1109 1110 1110 1110 1110 1110 1110 1110 1110 1110
1109 1110 1110 1110 1110 1110 1110 1110 1110 1110
1109 1109 1110 1110 1110 1110 1110 1110 1110 1110
1109 1109 1110 1110 1110 1110 1110 1110 1110 1110
1109 1109 1110 1110 1110 1110 1110 1110 1110 1110
Genomes with
BGC Present
1110.00
1109.75
1109.50
1109.25
1109.00

Fredericamycin

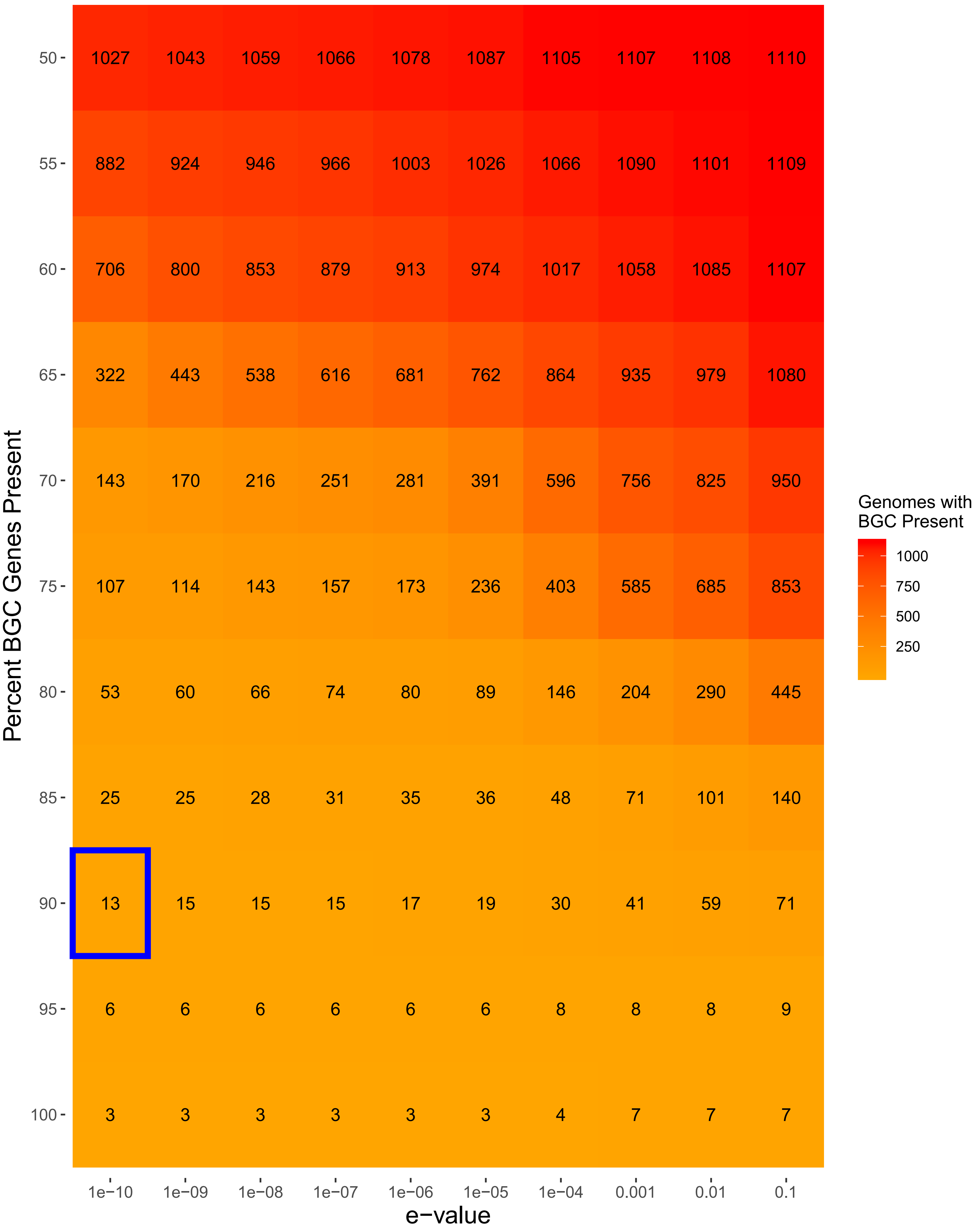

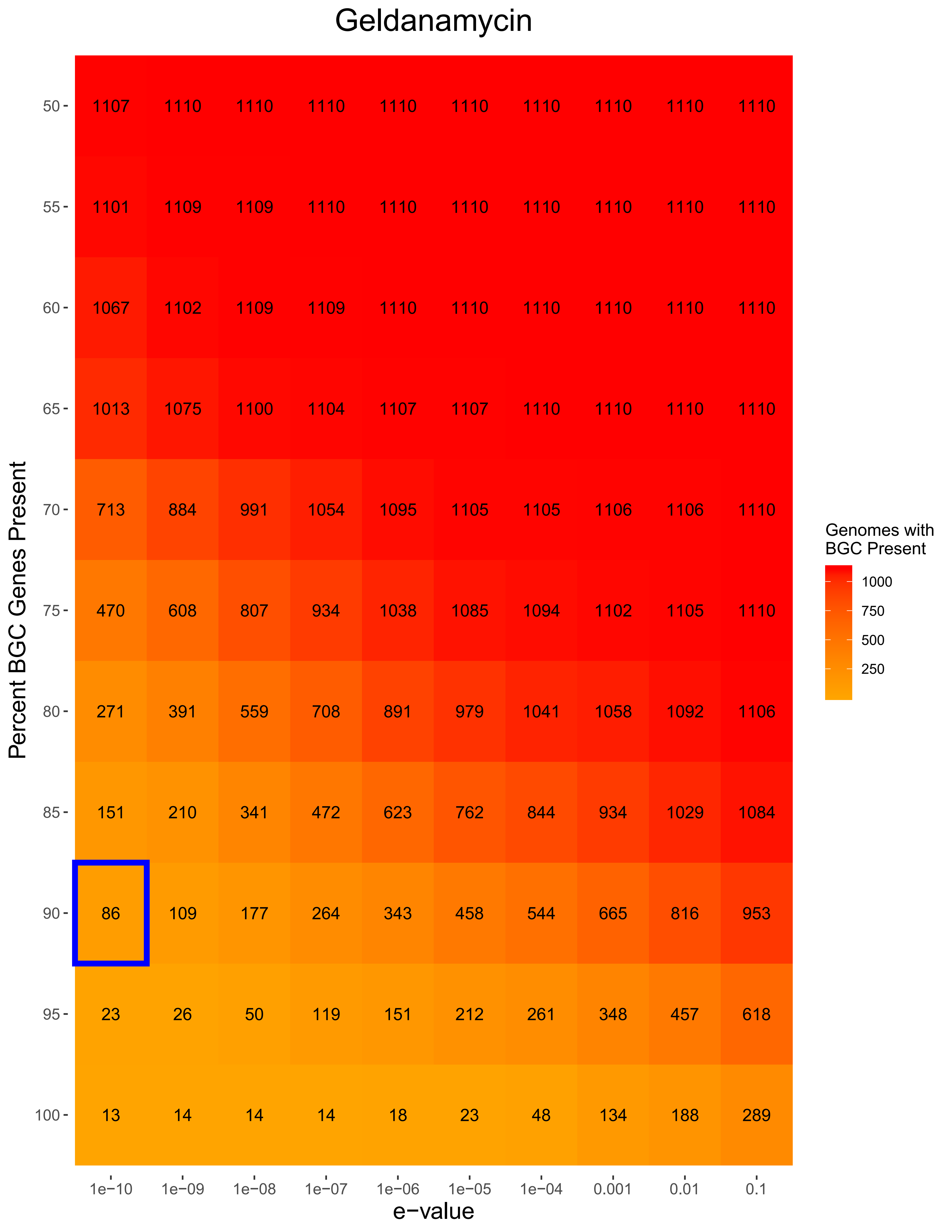
Geldanamycin
Percent BGC Genes Present
e−value
50
55
60
65
70
75
80
85
90
95
100
1e−10
1e−09
1e−08
1e−07
1e−06
1e−05
1e−04
0.001
0.01
0.1
1107 1110 1110 1110 1110 1110 1110 1110 1110 1110
1101 1109 1109 1110 1110 1110 1110 1110 1110 1110
1067 1102 1109 1109 1110 1110 1110 1110 1110 1110
1013 1075 1100 1104 1107 1107 1110 1110 1110 1110
713 884 991 1054 1095 1105 1105 1106 1106 1110
470 608 807 934 1038 1085 1094 1102 1105 1110
271 391 559 708 891 979 1041 1058 1092 1106
151 210 341 472 623 762 844 934 1029 1084
86 109 177 264 343 458 544 665 816 953
23 26 50 119 151 212 261 348 457 618
13 14 14 14 18 23 48 134 188 289
Genomes with BGC Present
1000
750
500
250

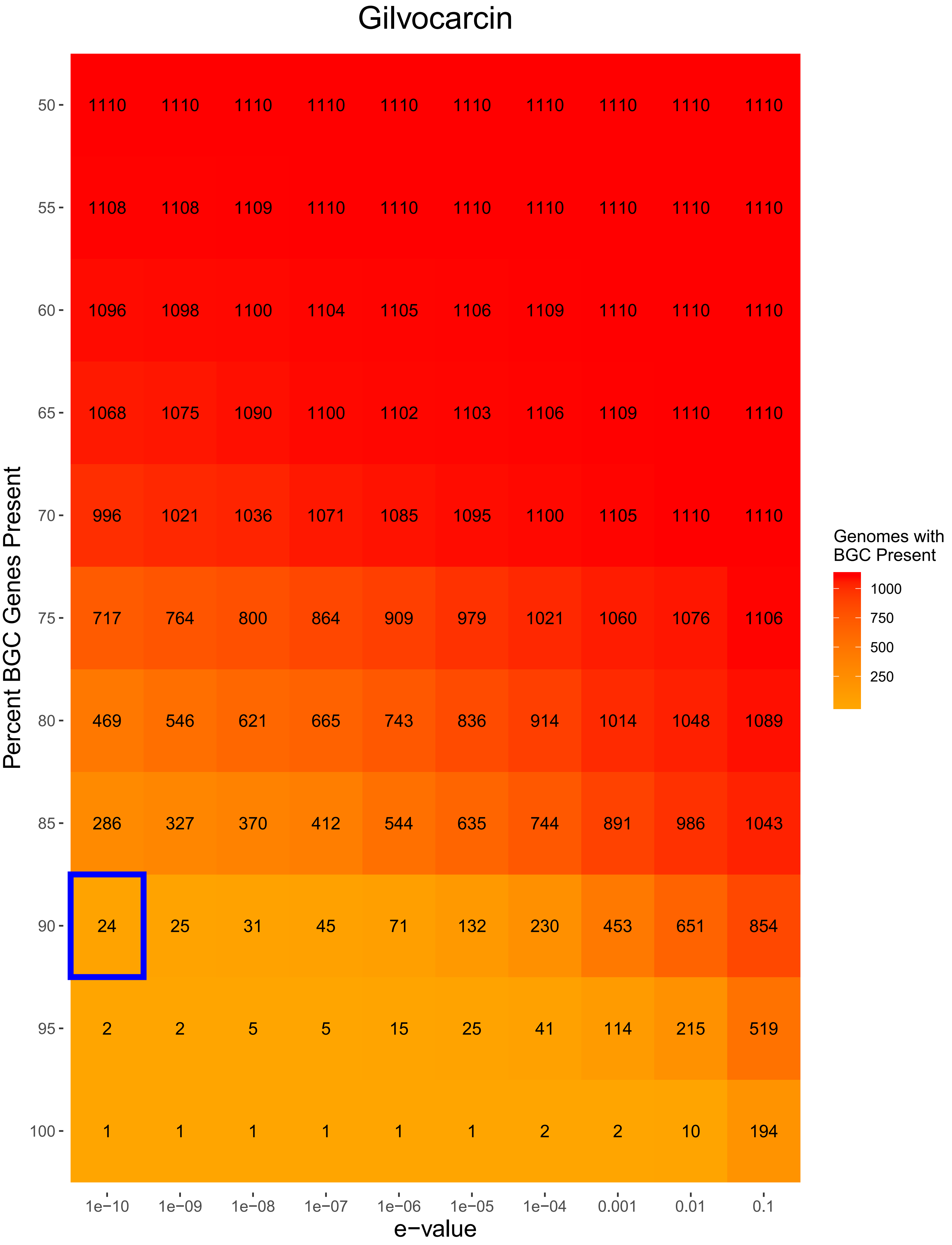
Gilvocarcin
Percent BGC Genes Present
e−value
Genomes with BGC Present
1000
750
500
250
1e−10 1e−09 1e−08 1e−07 1e−06 1e−05 1e−04 0.001 0.01 0.1
50 1110 1110 1110 1110 1110 1110 1110 1110 1110 1110
55 1108 1108 1109 1110 1110 1110 1110 1110 1110 1110
60 1096 1098 1100 1104 1105 1106 1109 1110 1110 1110
65 1068 1075 1090 1100 1102 1103 1106 1109 1110 1110
70 996 1021 1036 1071 1085 1095 1100 1105 1110 1110
75 717 764 800 864 909 979 1021 1060 1076 1106
80 469 546 621 665 743 836 914 1014 1048 1089
85 286 327 370 412 544 635 744 891 986 1043
90 24 25 31 45 71 132 230 453 651 854
95 2 2 5 5 15 25 41 114 215 519
100 1 1 1 1 1 1 2 2 10 194

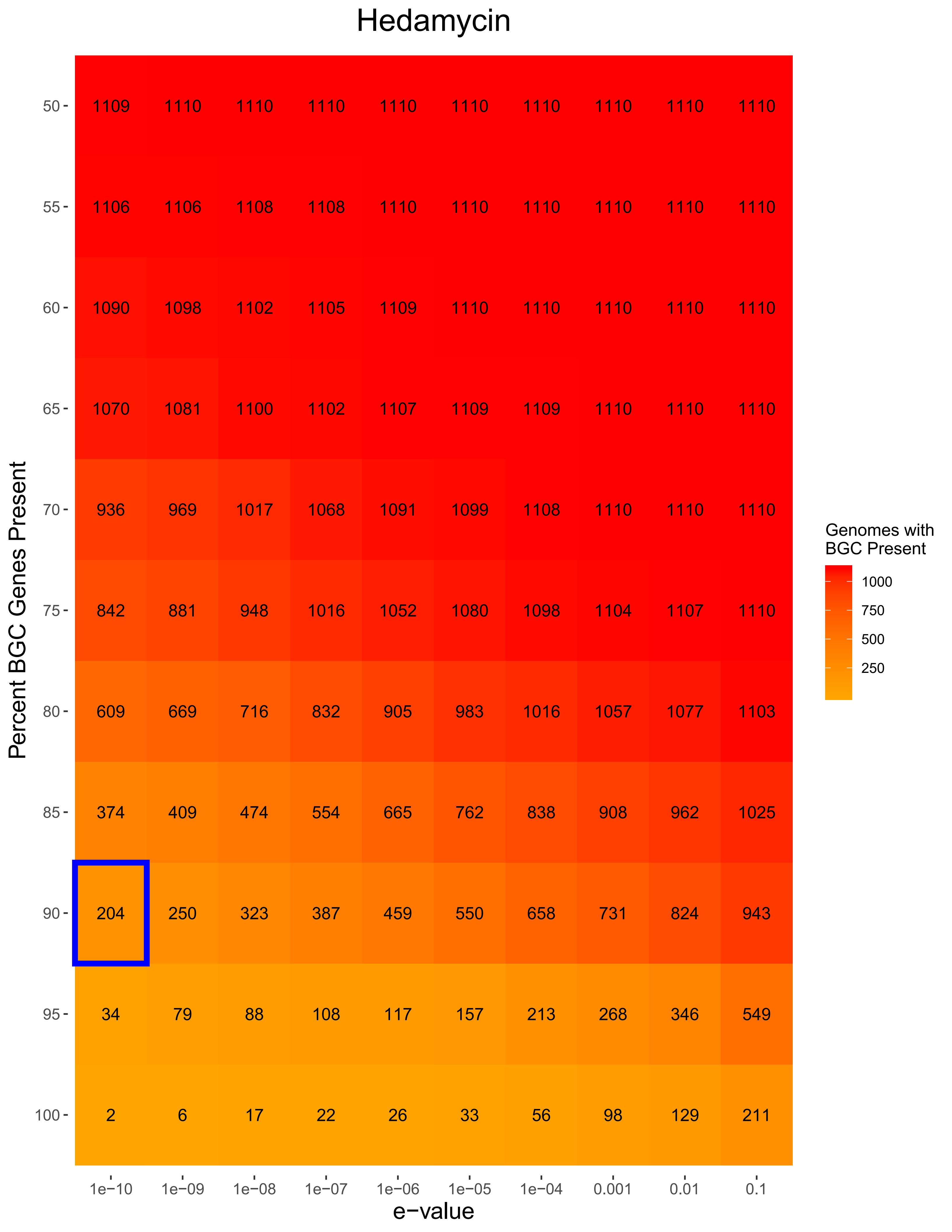

Hedamycin
Percent BGC Genes Present
e−value
Genomes with BGC Present
1000
750
500
250
50
55
60
65
70
75
80
85
90
95
100
1e−10
1e−09
1e−08
1e−07
1e−06
1e−05
1e−04
0.001
0.01
0.1
1109 1110 1110 1110 1110 1110 1110 1110 1110 1110
1106 1106 1108 1108 1110 1110 1110 1110 1110 1110
1090 1098 1102 1105 1109 1110 1110 1110 1110 1110
1070 1081 1100 1102 1107 1109 1109 1110 1110 1110
936 969 1017 1068 1091 1099 1108 1110 1110 1110
842 881 948 1016 1052 1080 1098 1104 1107 1110
609 669 716 832 905 983 1016 1057 1077 1103
374 409 474 554 665 762 838 908 962 1025
204 250 323 387 459 550 658 731 824 943
34 79 88 108 117 157 213 268 346 549
2 6 17 22 26 33 56 98 129 211

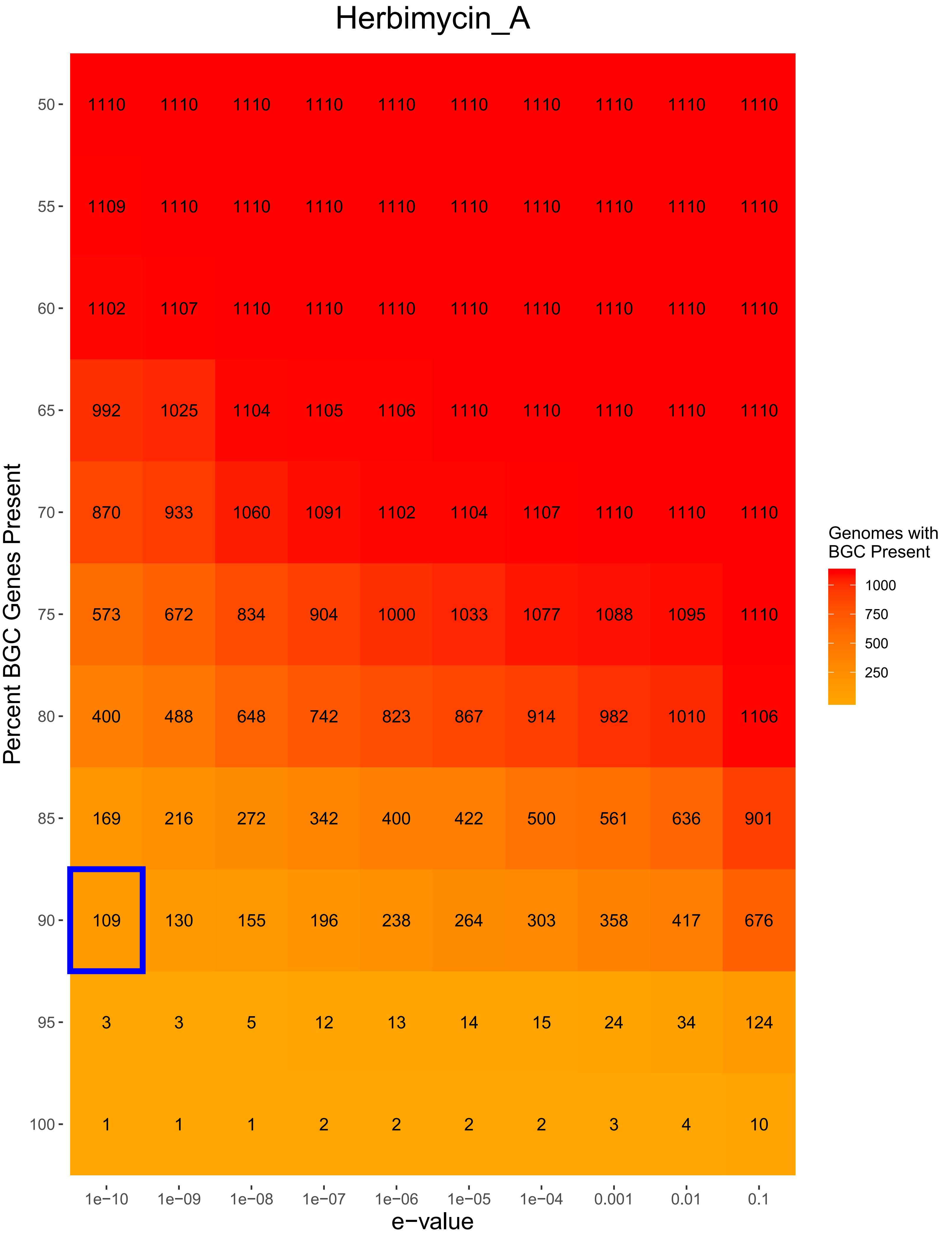

Herbimycin_A
Percent BGC Genes Present
e−value
1e−10
1e−09
1e−08
1e−07
1e−06
1e−05
1e−04
0.001
0.01
0.1
50
55
60
65
70
75
80
85
90
95
100
1110 1110 1110 1110 1110 1110 1110 1110 1110 1110
1109 1110 1110 1110 1110 1110 1110 1110 1110 1110
1102 1107 1110 1110 1110 1110 1110 1110 1110 1110
992 1025 1104 1105 1106 1110 1110 1110 1110 1110
870 933 1060 1091 1102 1104 1107 1110 1110 1110
573 672 834 904 1000 1033 1077 1088 1095 1110
400 488 648 742 823 867 914 982 1010 1106
169 216 272 342 400 422 500 561 636 901
109 130 155 196 238 264 303 358 417 676
3 3 5 12 13 14 15 24 34 124
1 1 1 2 2 2 2 3 4 10
Genomes with
BGC Present
1000
750
500
250

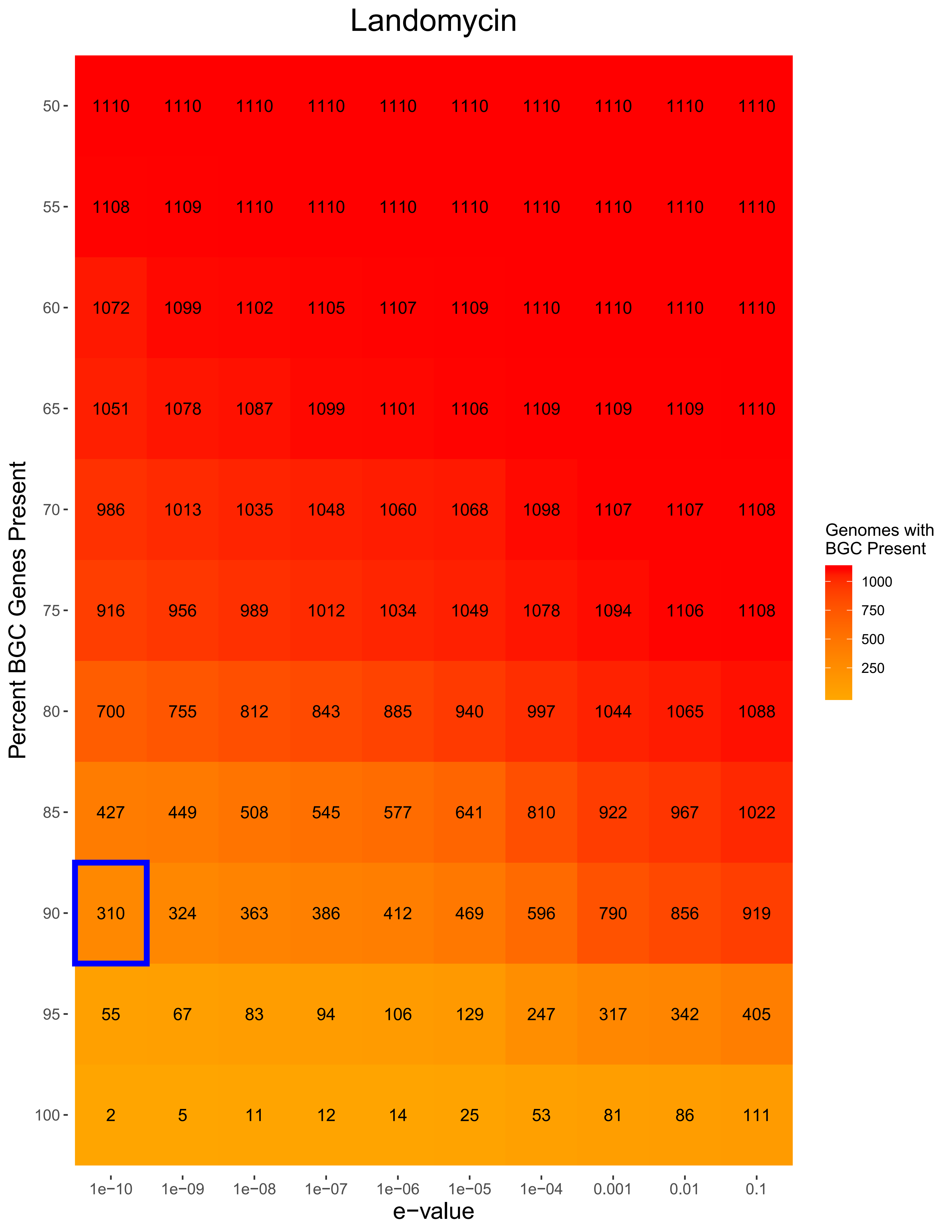
Landomycin
Percent BGC Genes Present
e−value
1e−10 1e−09 1e−08 1e−07 1e−06 1e−05 1e−04 0.001 0.01 0.1
50 55 60 65 70 75 80 85 90 95 100
50: 1110 1110 1110 1110 1110 1110 1110 1110 1110 1110
55: 1108 1109 1110 1110 1110 1110 1110 1110 1110 1110
60: 1072 1099 1102 1105 1107 1109 1110 1110 1110 1110
65: 1051 1078 1087 1099 1101 1106 1109 1109 1109 1110
70: 986 1013 1035 1048 1060 1068 1098 1107 1107 1108
75: 916 956 989 1012 1034 1049 1078 1094 1106 1108
80: 700 755 812 843 885 940 997 1044 1065 1088
85: 427 449 508 545 577 641 810 922 967 1022
90: 310 324 363 386 412 469 596 790 856 919
95: 55 67 83 94 106 129 247 317 342 405
100: 2 5 11 12 14 25 53 81 86 111
Genomes with BGC Present
1000
750
500
250

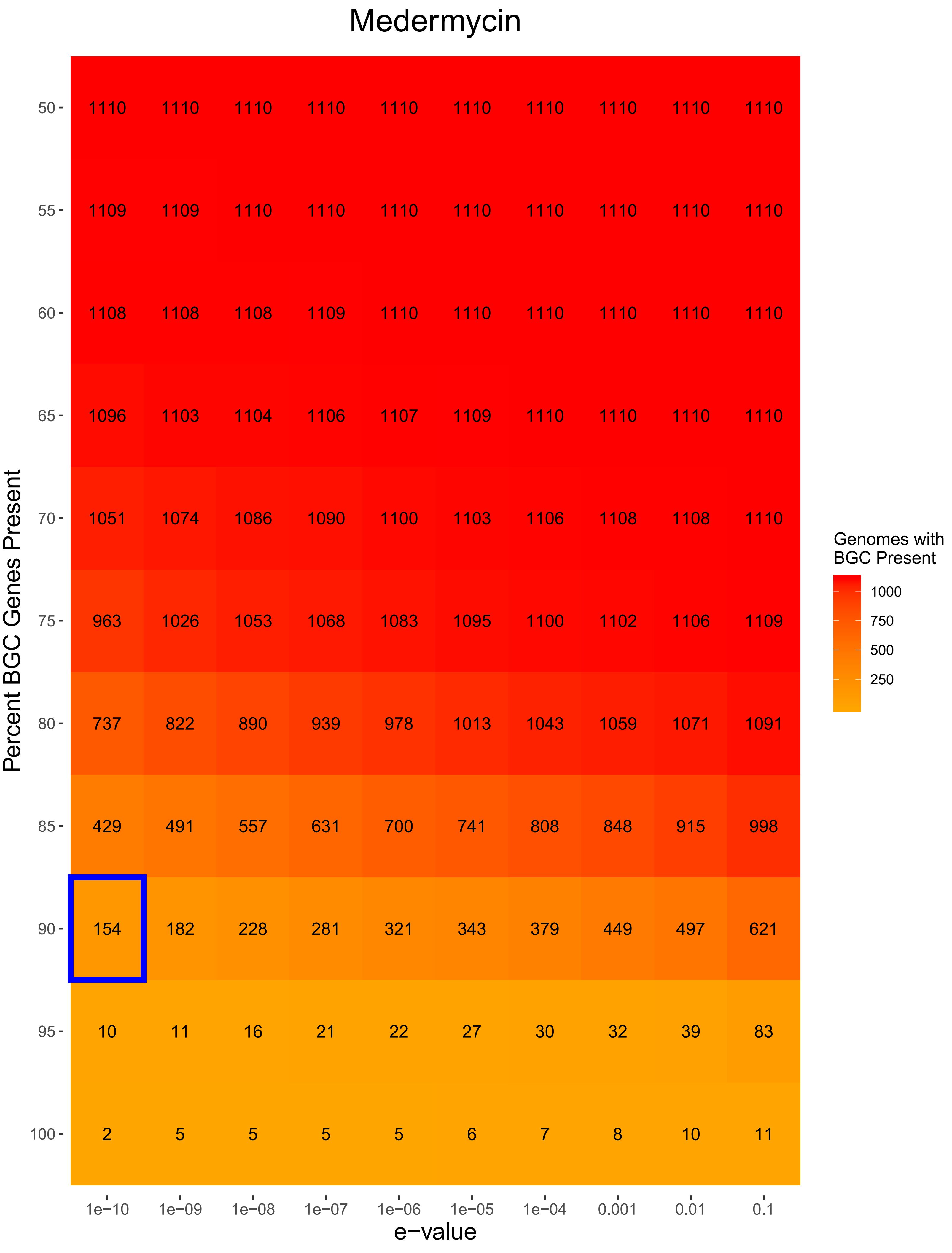
Medermycin
Percent BGC Genes Present
e−value
Genomes with BGC Present
1000
750
500
250
50 55 60 65 70 75 80 85 90 95 100
1e−10 1e−09 1e−08 1e−07 1e−06 1e−05 1e−04 0.001 0.01 0.1
50: 1110 1110 1110 1110 1110 1110 1110 1110 1110 1110
55: 1109 1109 1110 1110 1110 1110 1110 1110 1110 1110
60: 1108 1108 1108 1109 1110 1110 1110 1110 1110 1110
65: 1096 1103 1104 1106 1107 1109 1110 1110 1110 1110
70: 1051 1074 1086 1090 1100 1103 1106 1108 1108 1110
75: 963 1026 1053 1068 1083 1095 1100 1102 1106 1109
80: 737 822 890 939 978 1013 1043 1059 1071 1091
85: 429 491 557 631 700 741 808 848 915 998
90: 154 182 228 281 321 343 379 449 497 621
95: 10 11 16 21 22 27 30 32 39 83
100: 2 5 5 5 5 6 7 8 10 11

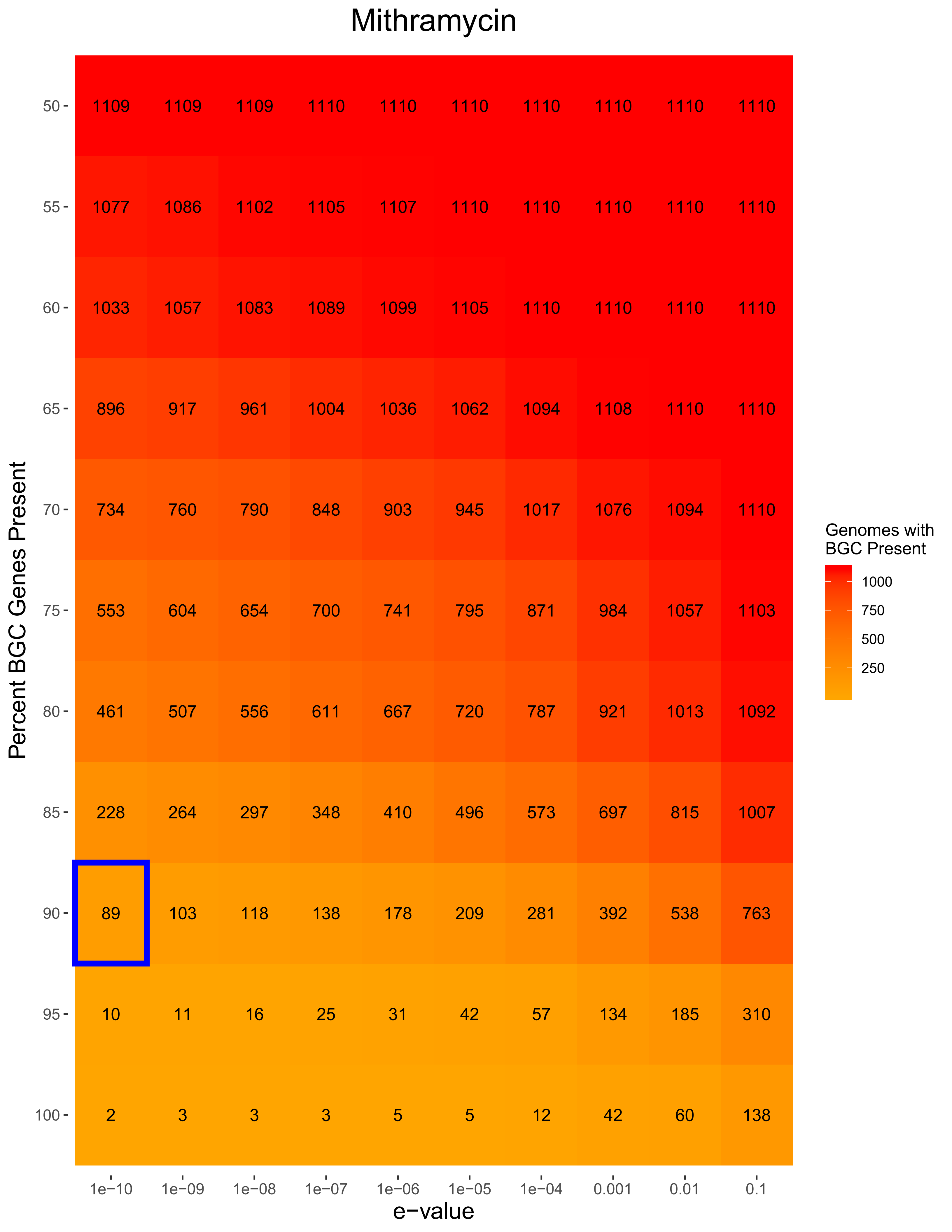
Mithramycin
Percent BGC Genes Present
e−value
1e−10 1e−09 1e−08 1e−07 1e−06 1e−05 1e−04 0.001 0.01 0.1
50 1109 1109 1109 1110 1110 1110 1110 1110 1110 1110
55 1077 1086 1102 1105 1107 1110 1110 1110 1110 1110
60 1033 1057 1083 1089 1099 1105 1110 1110 1110 1110
65 896 917 961 1004 1036 1062 1094 1108 1110 1110
70 734 760 790 848 903 945 1017 1076 1094 1110
75 553 604 654 700 741 795 871 984 1057 1103
80 461 507 556 611 667 720 787 921 1013 1092
85 228 264 297 348 410 496 573 697 815 1007
90 89 103 118 138 178 209 281 392 538 763
95 10 11 16 25 31 42 57 134 185 310
100 2 3 3 3 5 5 12 42 60 138
Genomes with BGC Present
1000
750
500
250

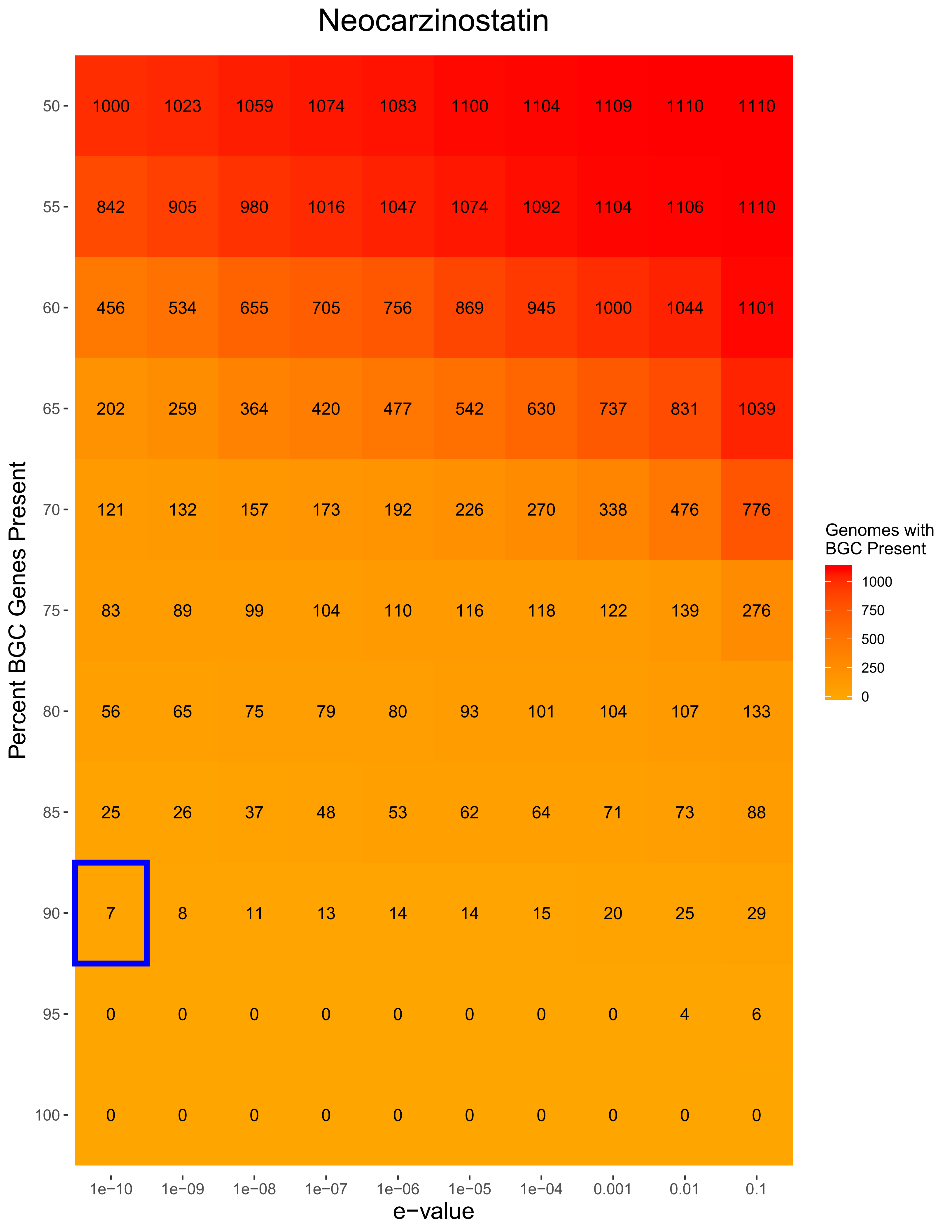
Neocarzinostatin
Percent BGC Genes Present
e−value
Genomes with BGC Present
1000
750
500
250
0
50 55 60 65 70 75 80 85 90 95 100
1e−10 1e−09 1e−08 1e−07 1e−06 1e−05 1e−04 0.001 0.01 0.1
1000 1023 1059 1074 1083 1100 1104 1109 1110 1110
842 905 980 1016 1047 1074 1092 1104 1106 1110
456 534 655 705 756 869 945 1000 1044 1101
202 259 364 420 477 542 630 737 831 1039
121 132 157 173 192 226 270 338 476 776
83 89 99 104 110 116 118 122 139 276
56 65 75 79 80 93 101 104 107 133
25 26 37 48 53 62 64 71 73 88
7 8 11 13 14 14 15 20 25 29
0 0 0 0 0 0 0 0 4 6
0 0 0 0 0 0 0 0 0 0

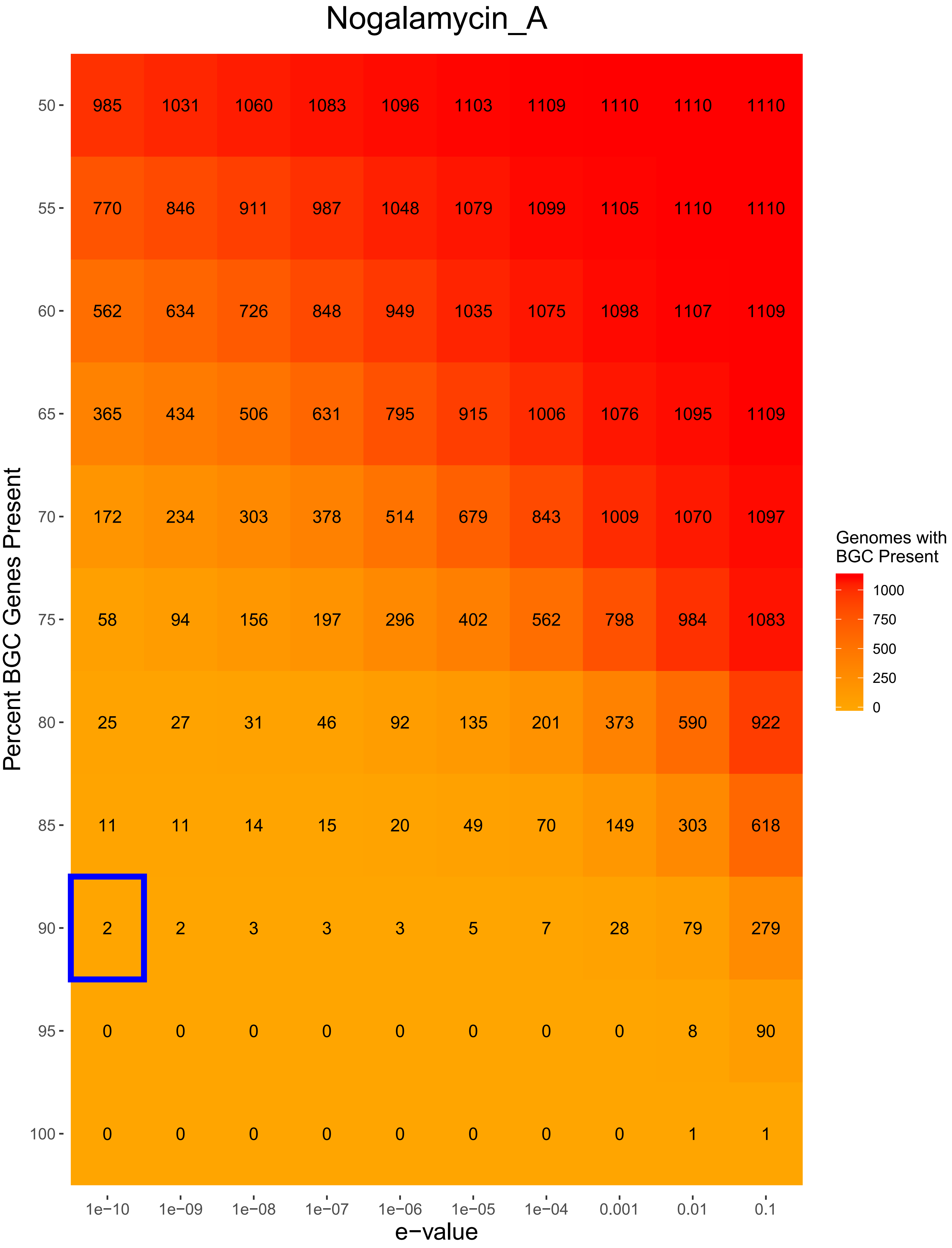

Nogalamycin_A
Percent BGC Genes Present
e−value
Genomes with BGC Present
1000
750
500
250
0
50
55
60
65
70
75
80
85
90
95
100
1e−10
1e−09
1e−08
1e−07
1e−06
1e−05
1e−04
0.001
0.01
0.1
985 1031 1060 1083 1096 1103 1109 1110 1110 1110
770 846 911 987 1048 1079 1099 1105 1110 1110
562 634 726 848 949 1035 1075 1098 1107 1109
365 434 506 631 795 915 1006 1076 1095 1109
172 234 303 378 514 679 843 1009 1070 1097
58 94 156 197 296 402 562 798 984 1083
25 27 31 46 92 135 201 373 590 922
11 11 14 15 20 49 70 149 303 618
2 2 3 3 3 5 7 28 79 279
0 0 0 0 0 0 0 0 8 90
0 0 0 0 0 0 0 0 1 1

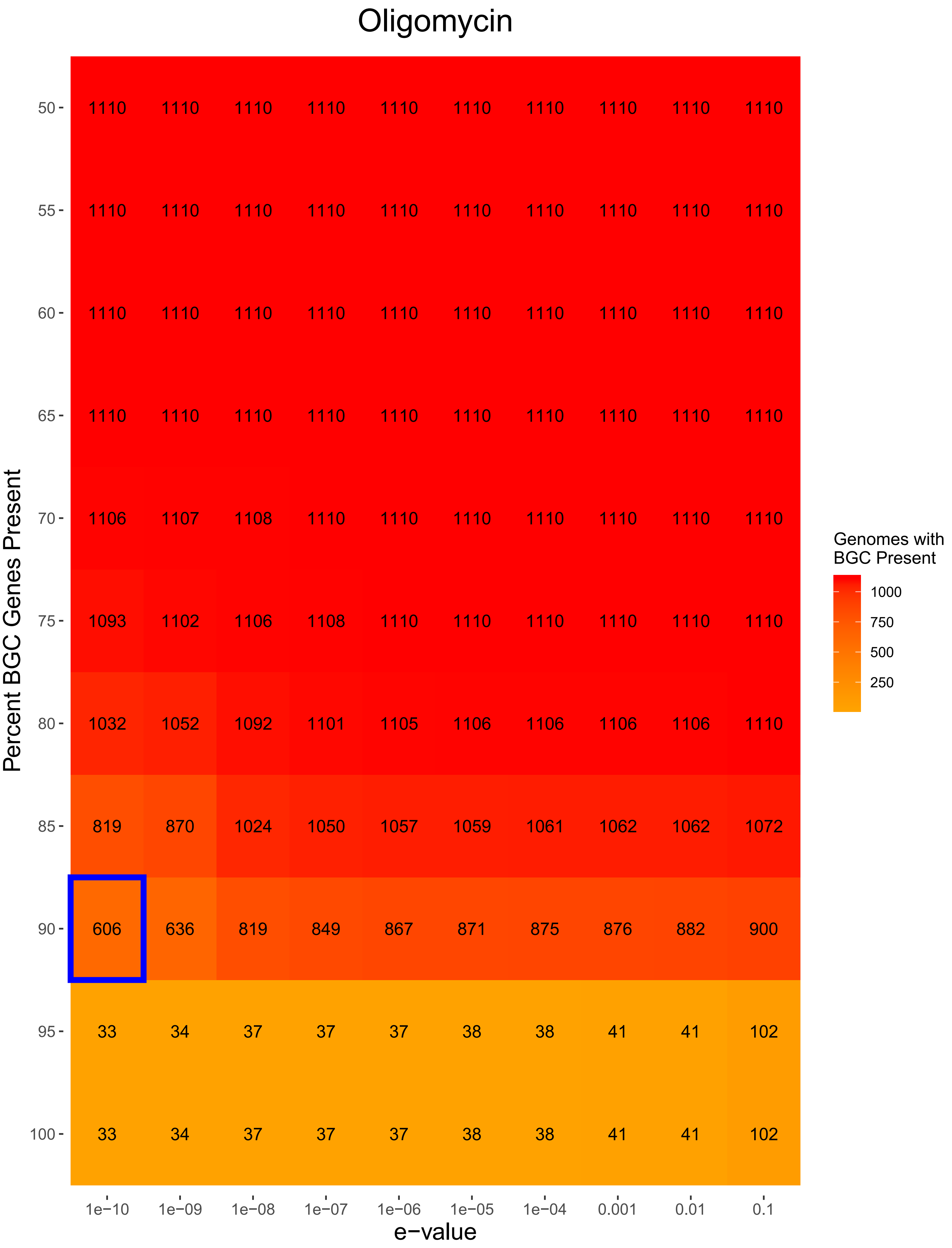
Oligomycin
Percent BGC Genes Present
e−value
Genomes with BGC Present
1000
750
500
250
50
55
60
65
70
75
80
85
90
95
100
1e−10
1e−09
1e−08
1e−07
1e−06
1e−05
1e−04
0.001
0.01
0.1
1110 1110 1110 1110 1110 1110 1110 1110 1110 1110
1110 1110 1110 1110 1110 1110 1110 1110 1110 1110
1110 1110 1110 1110 1110 1110 1110 1110 1110 1110
1110 1110 1110 1110 1110 1110 1110 1110 1110 1110
1106 1107 1108 1110 1110 1110 1110 1110 1110 1110
1093 1102 1106 1108 1110 1110 1110 1110 1110 1110
1032 1052 1092 1101 1105 1106 1106 1106 1106 1110
819 870 1024 1050 1057 1059 1061 1062 1062 1072
606 636 819 849 867 871 875 876 882 900
33 34 37 37 37 38 38 41 41 102
33 34 37 37 37 38 38 41 41 102

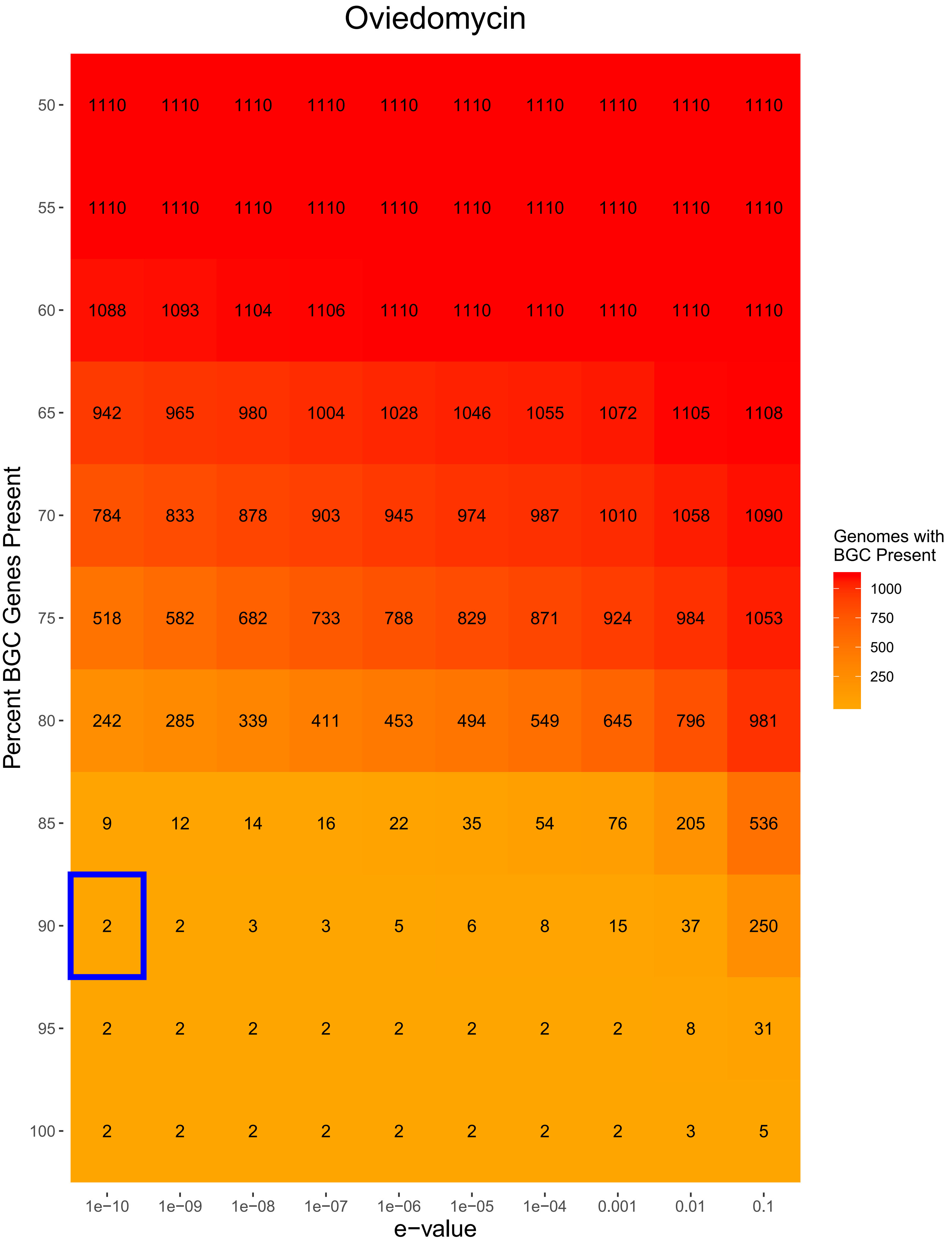
Oviedomycin
Percent BGC Genes Present
e−value
Genomes with BGC Present
1000
750
500
250
50
55
60
65
70
75
80
85
90
95
100
1e−10
1e−09
1e−08
1e−07
1e−06
1e−05
1e−04
0.001
0.01
0.1
1110 1110 1110 1110 1110 1110 1110 1110 1110 1110
1110 1110 1110 1110 1110 1110 1110 1110 1110 1110
1088 1093 1104 1106 1110 1110 1110 1110 1110 1110
942 965 980 1004 1028 1046 1055 1072 1105 1108
784 833 878 903 945 974 987 1010 1058 1090
518 582 682 733 788 829 871 924 984 1053
242 285 339 411 453 494 549 645 796 981
9 12 14 16 22 35 54 76 205 536
2 2 3 3 5 6 8 15 37 250
2 2 2 2 2 2 2 2 8 31
2 2 2 2 2 2 2 2 3 5

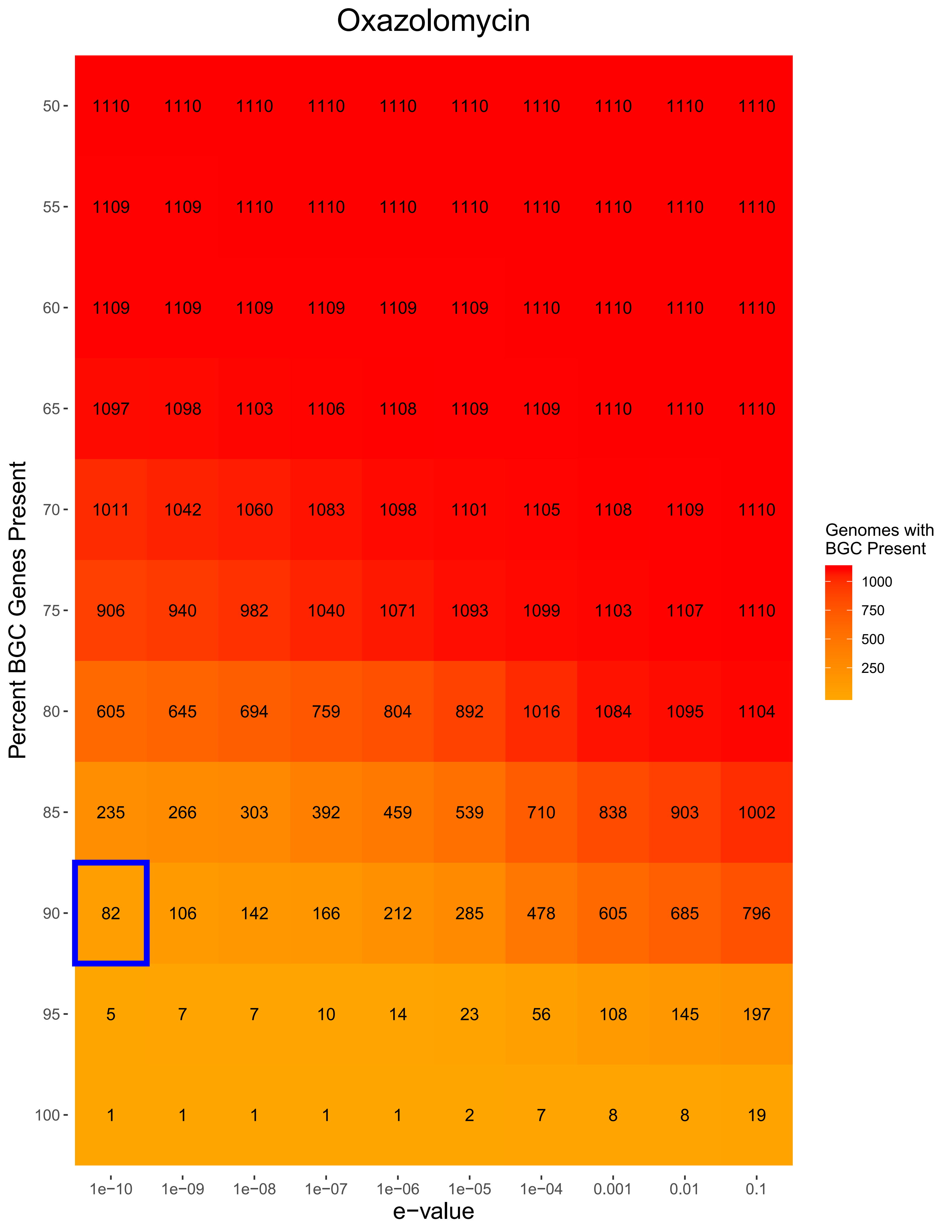
Oxazolomycin
Percent BGC Genes Present
e−value
Genomes with BGC Present
1000
750
500
250
50
55
60
65
70
75
80
85
90
95
100
1e−10
1e−09
1e−08
1e−07
1e−06
1e−05
1e−04
0.001
0.01
0.1
1110 1110 1110 1110 1110 1110 1110 1110 1110 1110
1109 1109 1110 1110 1110 1110 1110 1110 1110 1110
1109 1109 1109 1109 1109 1109 1110 1110 1110 1110
1097 1098 1103 1106 1108 1109 1109 1110 1110 1110
1011 1042 1060 1083 1098 1101 1105 1108 1109 1110
906 940 982 1040 1071 1093 1099 1103 1107 1110
605 645 694 759 804 892 1016 1084 1095 1104
235 266 303 392 459 539 710 838 903 1002
82 106 142 166 212 285 478 605 685 796
5 7 7 10 14 23 56 108 145 197
1 1 1 1 1 2 7 8 8 19

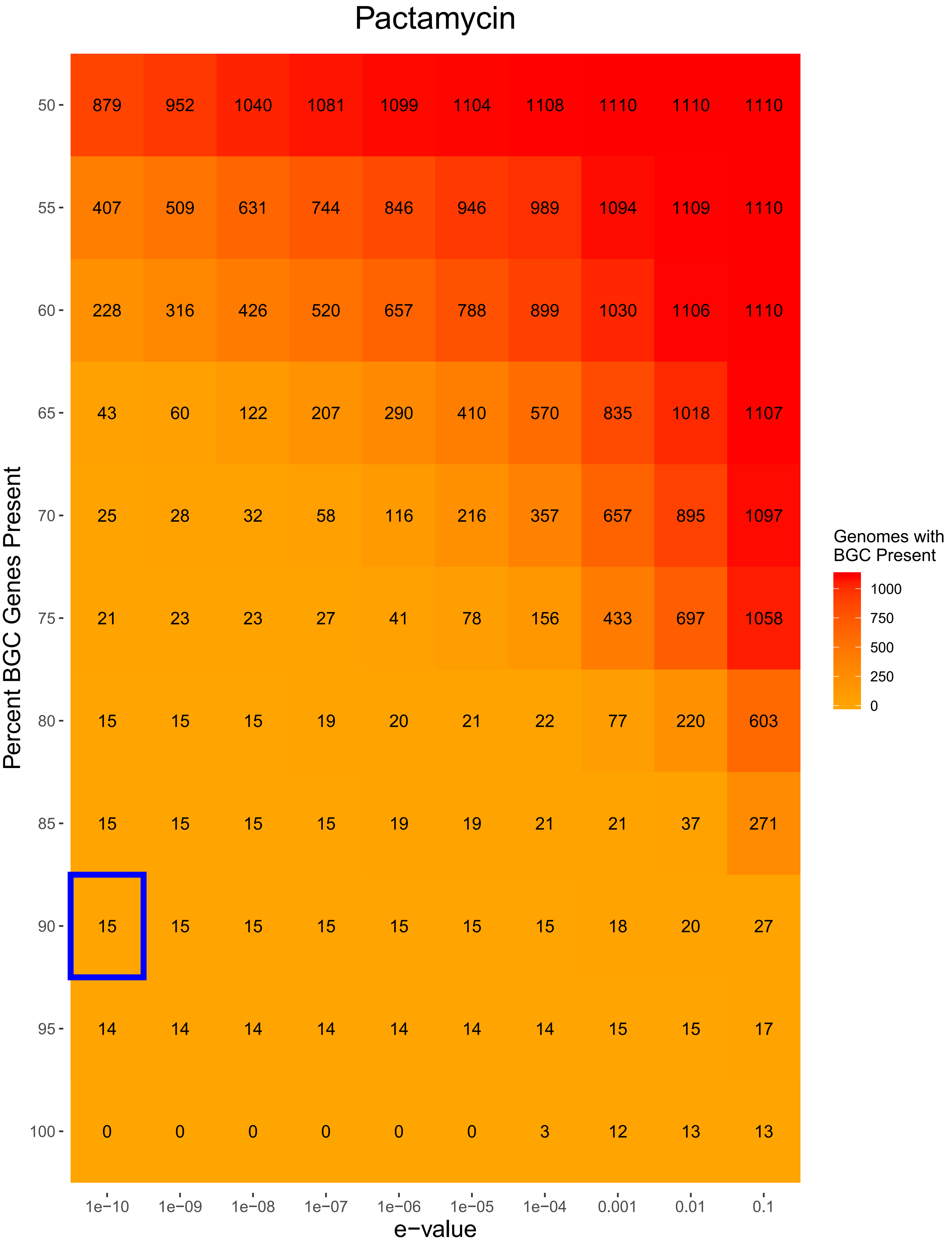
Pactamycin
Percent BGC Genes Present
e−value
Genomes with BGC Present
1000
750
500
250
0
50
55
60
65
70
75
80
85
90
95
100
1e−10
1e−09
1e−08
1e−07
1e−06
1e−05
1e−04
0.001
0.01
0.1
879 952 1040 1081 1099 1104 1108 1110 1110 1110
407 509 631 744 846 946 989 1094 1109 1110
228 316 426 520 657 788 899 1030 1106 1110
43 60 122 207 290 410 570 835 1018 1107
25 28 32 58 116 216 357 657 895 1097
21 23 23 27 41 78 156 433 697 1058
15 15 15 19 20 21 22 77 220 603
15 15 15 15 19 19 21 21 37 271
15 15 15 15 15 15 15 18 20 27
14 14 14 14 14 14 14 15 15 17
0 0 0 0 0 0 3 12 13 13

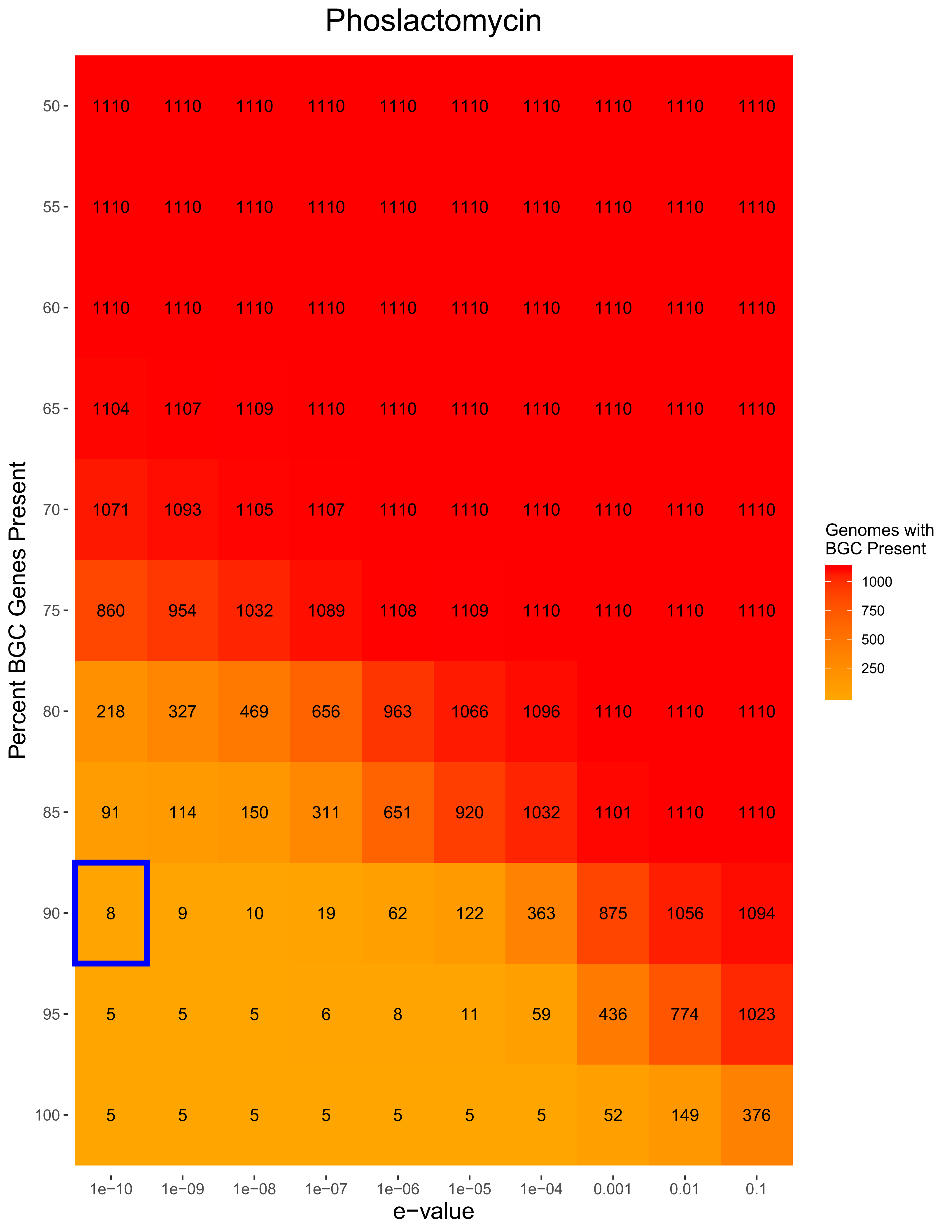

Phoslactomycin
Percent BGC Genes Present
e−value
Genomes with BGC Present
1000
750
500
250
50
55
60
65
70
75
80
85
90
95
100
1e−10
1e−09
1e−08
1e−07
1e−06
1e−05
1e−04
0.001
0.01
0.1
1110 1110 1110 1110 1110 1110 1110 1110 1110 1110
1110 1110 1110 1110 1110 1110 1110 1110 1110 1110
1110 1110 1110 1110 1110 1110 1110 1110 1110 1110
1104 1107 1109 1110 1110 1110 1110 1110 1110 1110
1071 1093 1105 1107 1110 1110 1110 1110 1110 1110
860 954 1032 1089 1108 1109 1110 1110 1110 1110
218 327 469 656 963 1066 1096 1110 1110 1110
91 114 150 311 651 920 1032 1101 1110 1110
8 9 10 19 62 122 363 875 1056 1094
5 5 5 6 8 11 59 436 774 1023
5 5 5 5 5 5 5 52 149 376

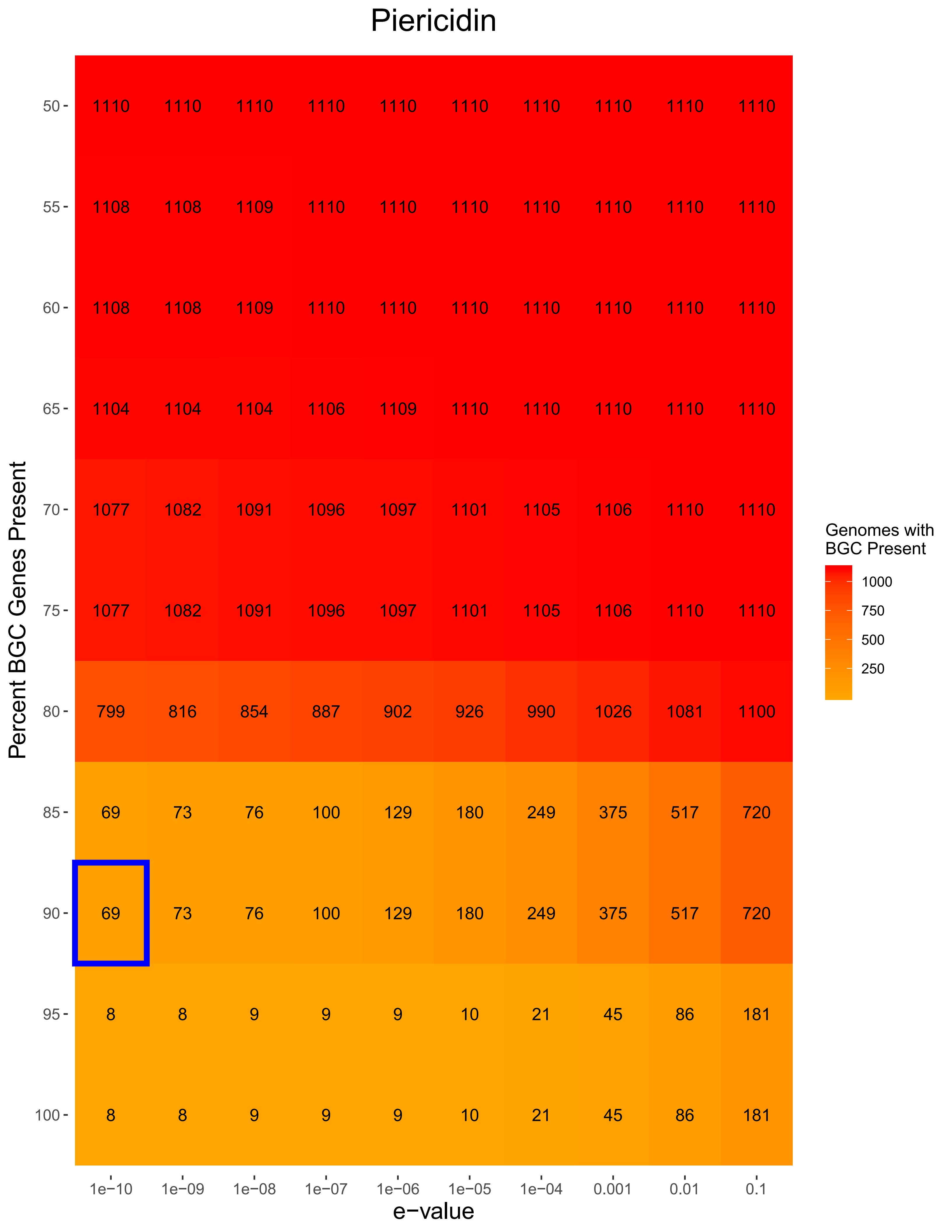

Piericidin
Percent BGC Genes Present
e−value
Genomes with BGC Present
1000
750
500
250
50
55
60
65
70
75
80
85
90
95
100
1e−10
1e−09
1e−08
1e−07
1e−06
1e−05
1e−04
0.001
0.01
0.1
1110 1110 1110 1110 1110 1110 1110 1110 1110 1110
1108 1108 1109 1110 1110 1110 1110 1110 1110 1110
1108 1108 1109 1110 1110 1110 1110 1110 1110 1110
1104 1104 1104 1106 1109 1110 1110 1110 1110 1110
1077 1082 1091 1096 1097 1101 1105 1106 1110 1110
1077 1082 1091 1096 1097 1101 1105 1106 1110 1110
799 816 854 887 902 926 990 1026 1081 1100
69 73 76 100 129 180 249 375 517 720
69 73 76 100 129 180 249 375 517 720
8 8 9 9 9 10 21 45 86 181
8 8 9 9 9 10 21 45 86 181

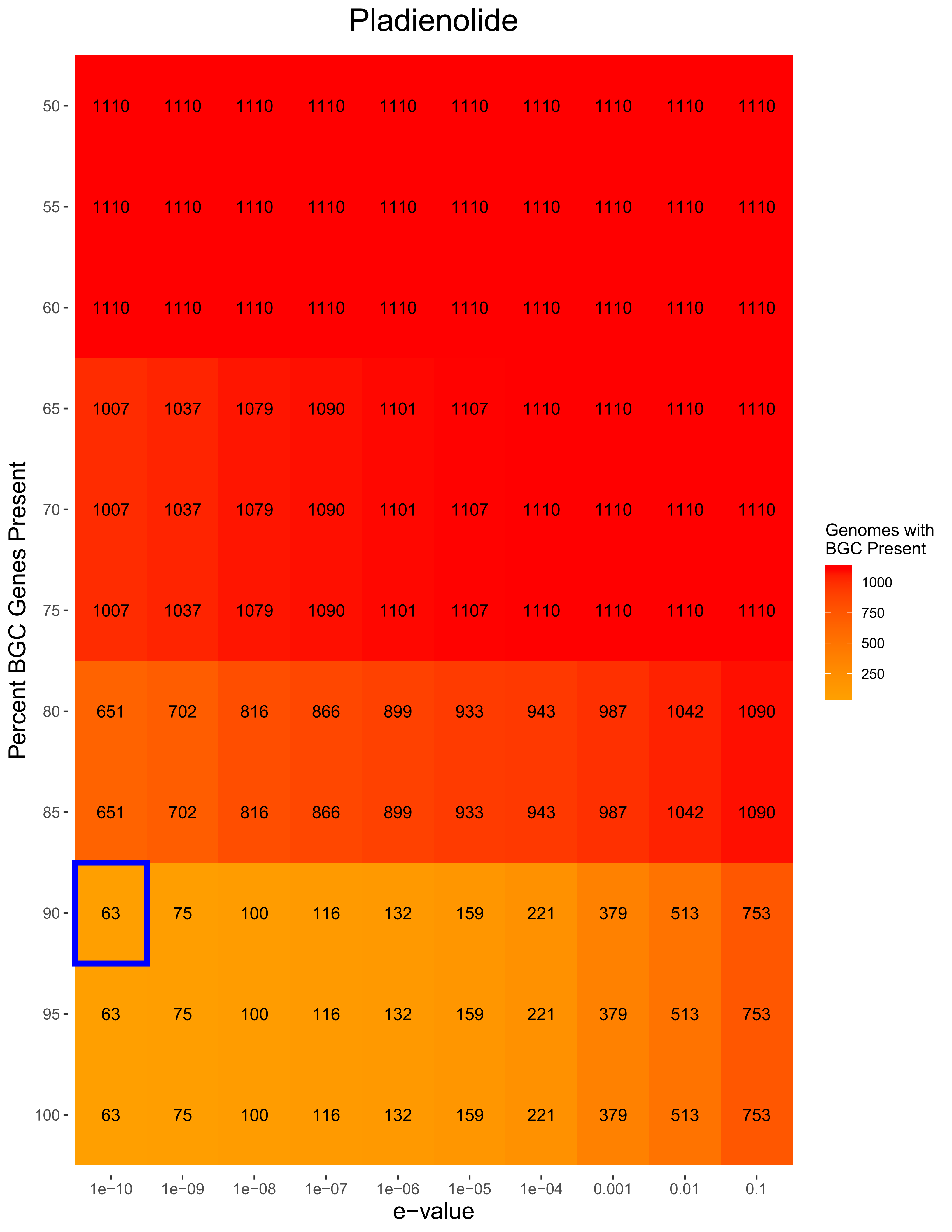
Pladienolide
Percent BGC Genes Present
e−value
1e−10
1e−09
1e−08
1e−07
1e−06
1e−05
1e−04
0.001
0.01
0.1
50
55
60
65
70
75
80
85
90
95
100
1110 1110 1110 1110 1110 1110 1110 1110 1110 1110
1110 1110 1110 1110 1110 1110 1110 1110 1110 1110
1110 1110 1110 1110 1110 1110 1110 1110 1110 1110
1007 1037 1079 1090 1101 1107 1110 1110 1110 1110
1007 1037 1079 1090 1101 1107 1110 1110 1110 1110
1007 1037 1079 1090 1101 1107 1110 1110 1110 1110
651 702 816 866 899 933 943 987 1042 1090
651 702 816 866 899 933 943 987 1042 1090
63 75 100 116 132 159 221 379 513 753
63 75 100 116 132 159 221 379 513 753
63 75 100 116 132 159 221 379 513 753
Genomes with
BGC Present
1000
750
500
250

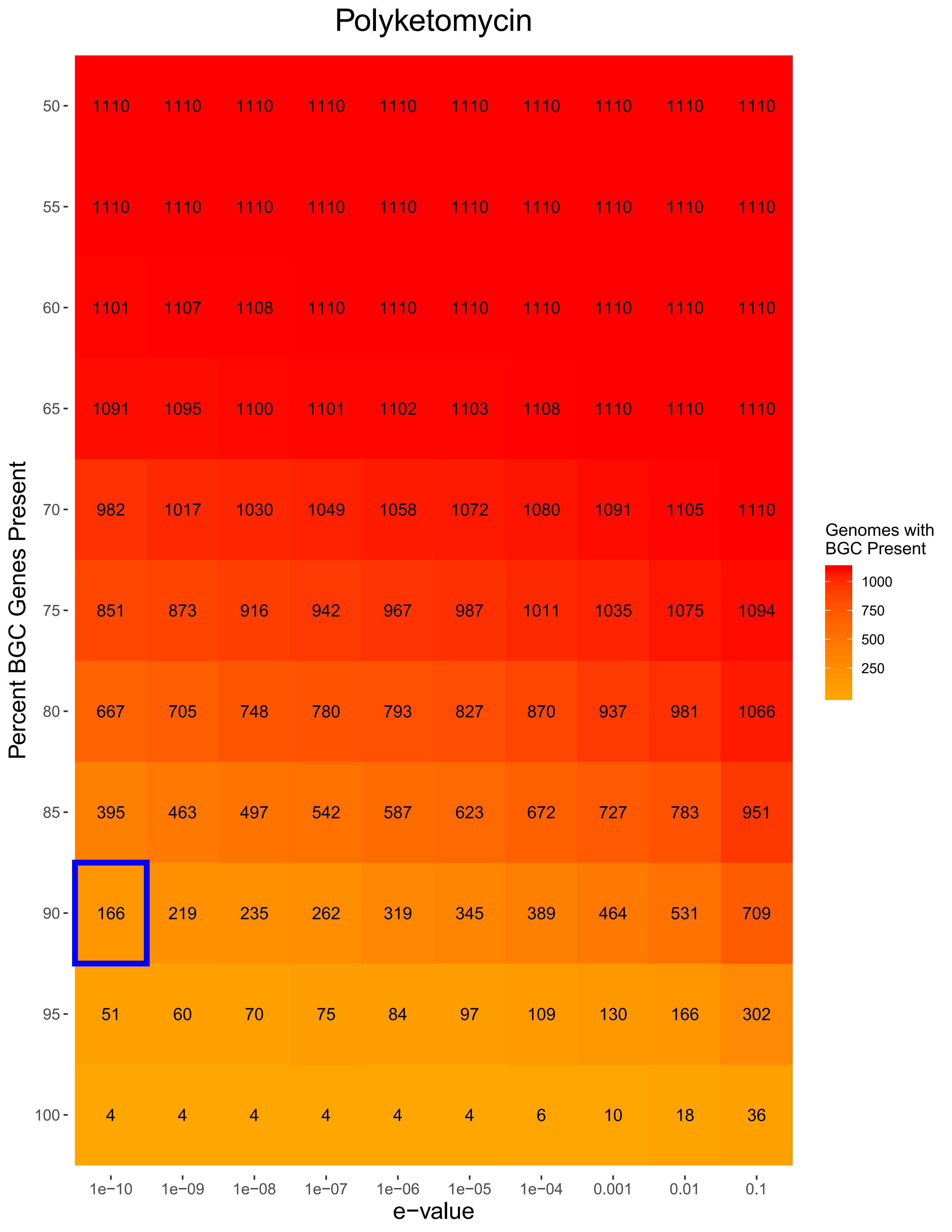

Polyketomycin
Percent BGC Genes Present
e−value
Genomes with BGC Present
1000
750
500
250
50
55
60
65
70
75
80
85
90
95
100
1e−10
1e−09
1e−08
1e−07
1e−06
1e−05
1e−04
0.001
0.01
0.1
1110 1110 1110 1110 1110 1110 1110 1110 1110 1110
1110 1110 1110 1110 1110 1110 1110 1110 1110 1110
1101 1107 1108 1110 1110 1110 1110 1110 1110 1110
1091 1095 1100 1101 1102 1103 1108 1110 1110 1110
982 1017 1030 1049 1058 1072 1080 1091 1105 1110
851 873 916 942 967 987 1011 1035 1075 1094
667 705 748 780 793 827 870 937 981 1066
395 463 497 542 587 623 672 727 783 951
166 219 235 262 319 345 389 464 531 709
51 60 70 75 84 97 109 130 166 302
4 4 4 4 4 4 6 10 18 36

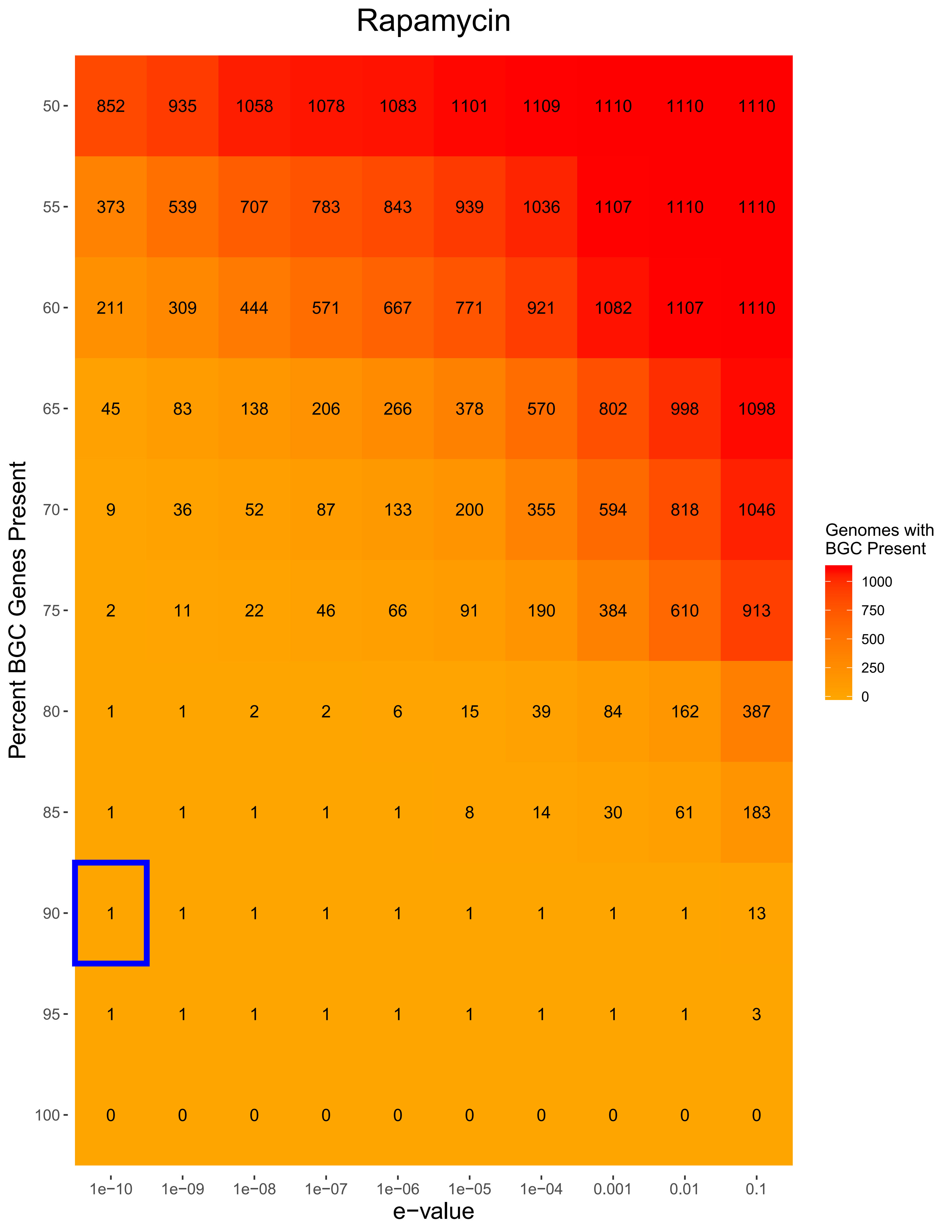
Rapamycin
Percent BGC Genes Present
e−value
Genomes with BGC Present
1000
750
500
250
0
50 55 60 65 70 75 80 85 90 95 100
1e−10 1e−09 1e−08 1e−07 1e−06 1e−05 1e−04 0.001 0.01 0.1
852 935 1058 1078 1083 1101 1109 1110 1110 1110
373 539 707 783 843 939 1036 1107 1110 1110
211 309 444 571 667 771 921 1082 1107 1110
45 83 138 206 266 378 570 802 998 1098
9 36 52 87 133 200 355 594 818 1046
2 11 22 46 66 91 190 384 610 913
1 1 2 2 6 15 39 84 162 387
1 1 1 1 1 8 14 30 61 183
1 1 1 1 1 1 1 1 1 13
1 1 1 1 1 1 1 1 1 3
0 0 0 0 0 0 0 0 0 0

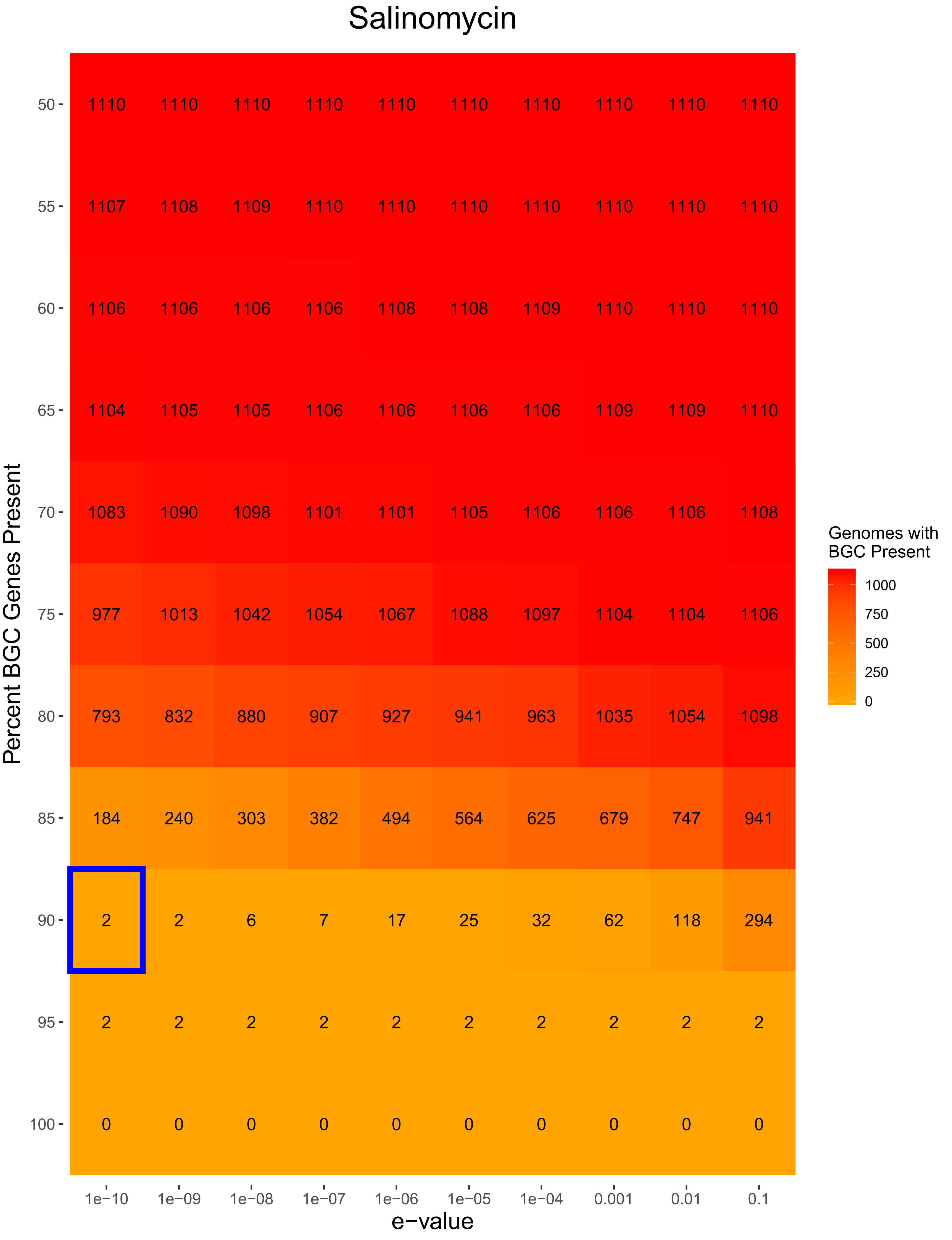
Salinomycin
Percent BGC Genes Present
e−value
Genomes with BGC Present
1e−10 1e−09 1e−08 1e−07 1e−06 1e−05 1e−04 0.001 0.01 0.1
50 55 60 65 70 75 80 85 90 95 100
0 250 500 750 1000
50: 1110 1110 1110 1110 1110 1110 1110 1110 1110 1110
55: 1107 1108 1109 1110 1110 1110 1110 1110 1110 1110
60: 1106 1106 1106 1106 1108 1108 1109 1110 1110 1110
65: 1104 1105 1105 1106 1106 1106 1106 1109 1109 1110
70: 1083 1090 1098 1101 1101 1105 1106 1106 1106 1108
75: 977 1013 1042 1054 1067 1088 1097 1104 1104 1106
80: 793 832 880 907 927 941 963 1035 1054 1098
85: 184 240 303 382 494 564 625 679 747 941
90: 2 2 6 7 17 25 32 62 118 294
95: 2 2 2 2 2 2 2 2 2 2
100: 0 0 0 0 0 0 0 0 0 0

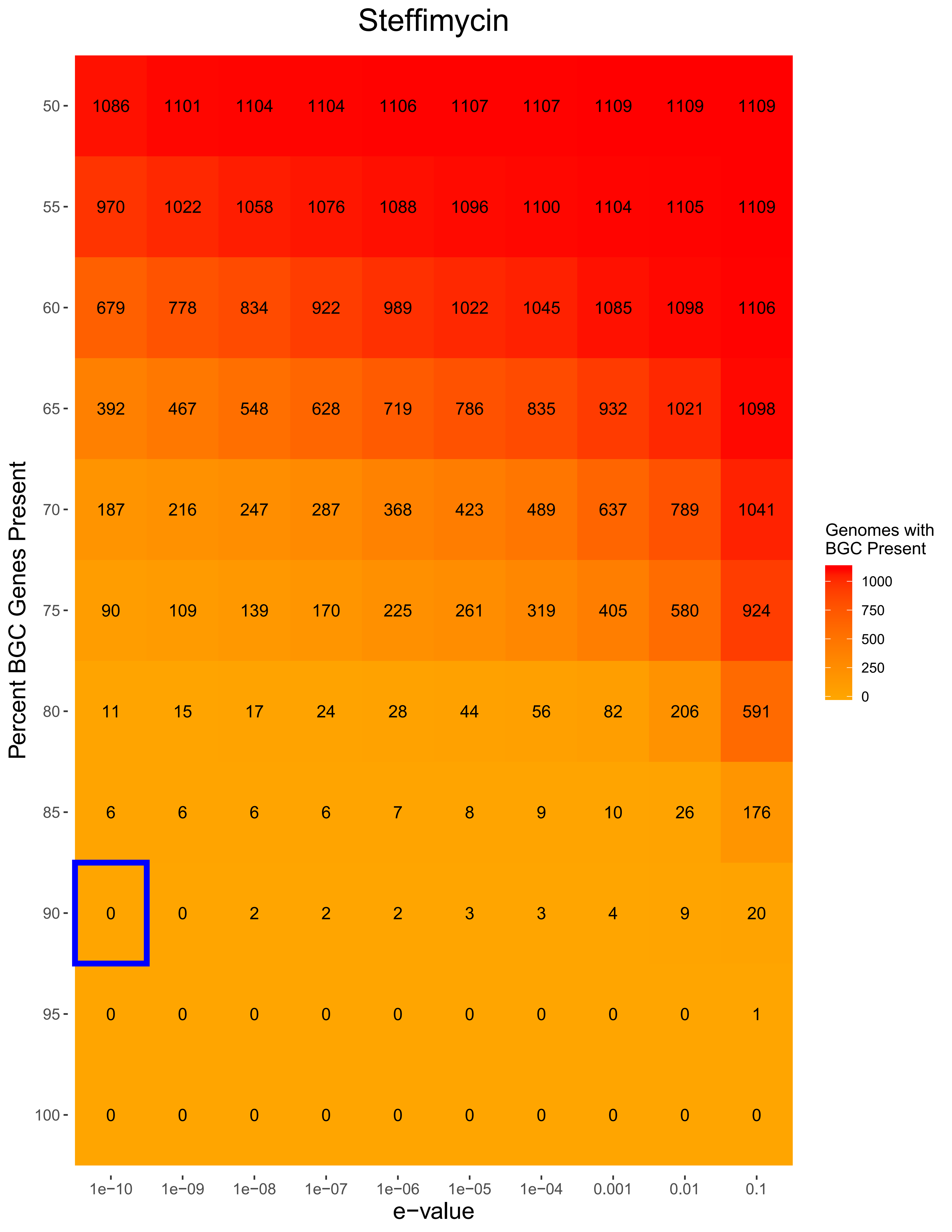
Steffimycin
Percent BGC Genes Present
e−value
Genomes with BGC Present
1000
750
500
250
0
50
55
60
65
70
75
80
85
90
95
100
1e−10
1e−09
1e−08
1e−07
1e−06
1e−05
1e−04
0.001
0.01
0.1
1086 1101 1104 1104 1106 1107 1107 1109 1109 1109
970 1022 1058 1076 1088 1096 1100 1104 1105 1109
679 778 834 922 989 1022 1045 1085 1098 1106
392 467 548 628 719 786 835 932 1021 1098
187 216 247 287 368 423 489 637 789 1041
90 109 139 170 225 261 319 405 580 924
11 15 17 24 28 44 56 82 206 591
6 6 6 6 7 8 9 10 26 176
0 0 2 2 2 3 3 4 9 20
0 0 0 0 0 0 0 0 0 1
0 0 0 0 0 0 0 0 0 0

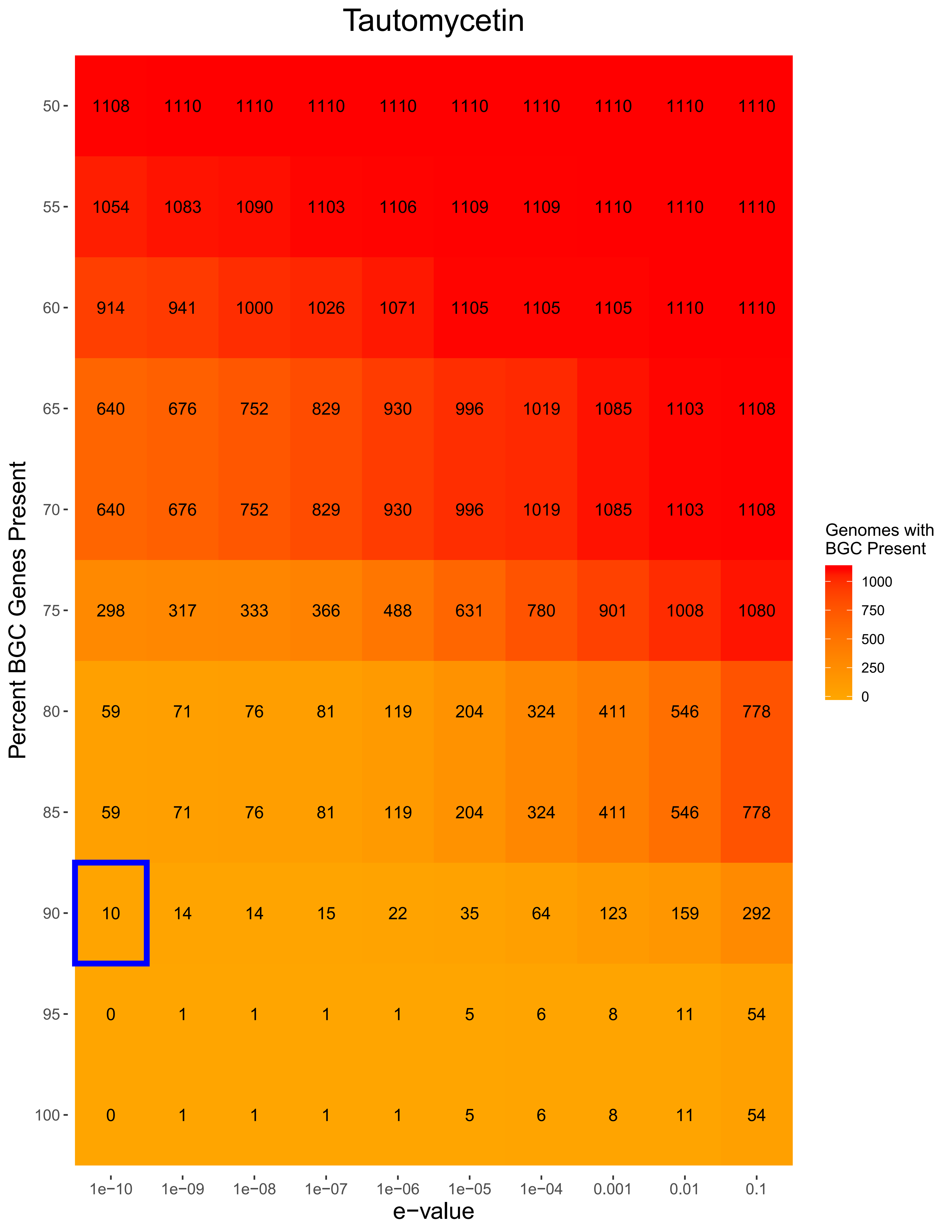

Tautomycetin
Percent BGC Genes Present
e−value
Genomes with BGC Present
1000
750
500
250
0
50
55
60
65
70
75
80
85
90
95
100
1e−10
1e−09
1e−08
1e−07
1e−06
1e−05
1e−04
0.001
0.01
0.1
1108 1110 1110 1110 1110 1110 1110 1110 1110 1110
1054 1083 1090 1103 1106 1109 1109 1110 1110 1110
914 941 1000 1026 1071 1105 1105 1105 1110 1110
640 676 752 829 930 996 1019 1085 1103 1108
640 676 752 829 930 996 1019 1085 1103 1108
298 317 333 366 488 631 780 901 1008 1080
59 71 76 81 119 204 324 411 546 778
59 71 76 81 119 204 324 411 546 778
10 14 14 15 22 35 64 123 159 292
0 1 1 1 1 5 6 8 11 54
0 1 1 1 1 5 6 8 11 54

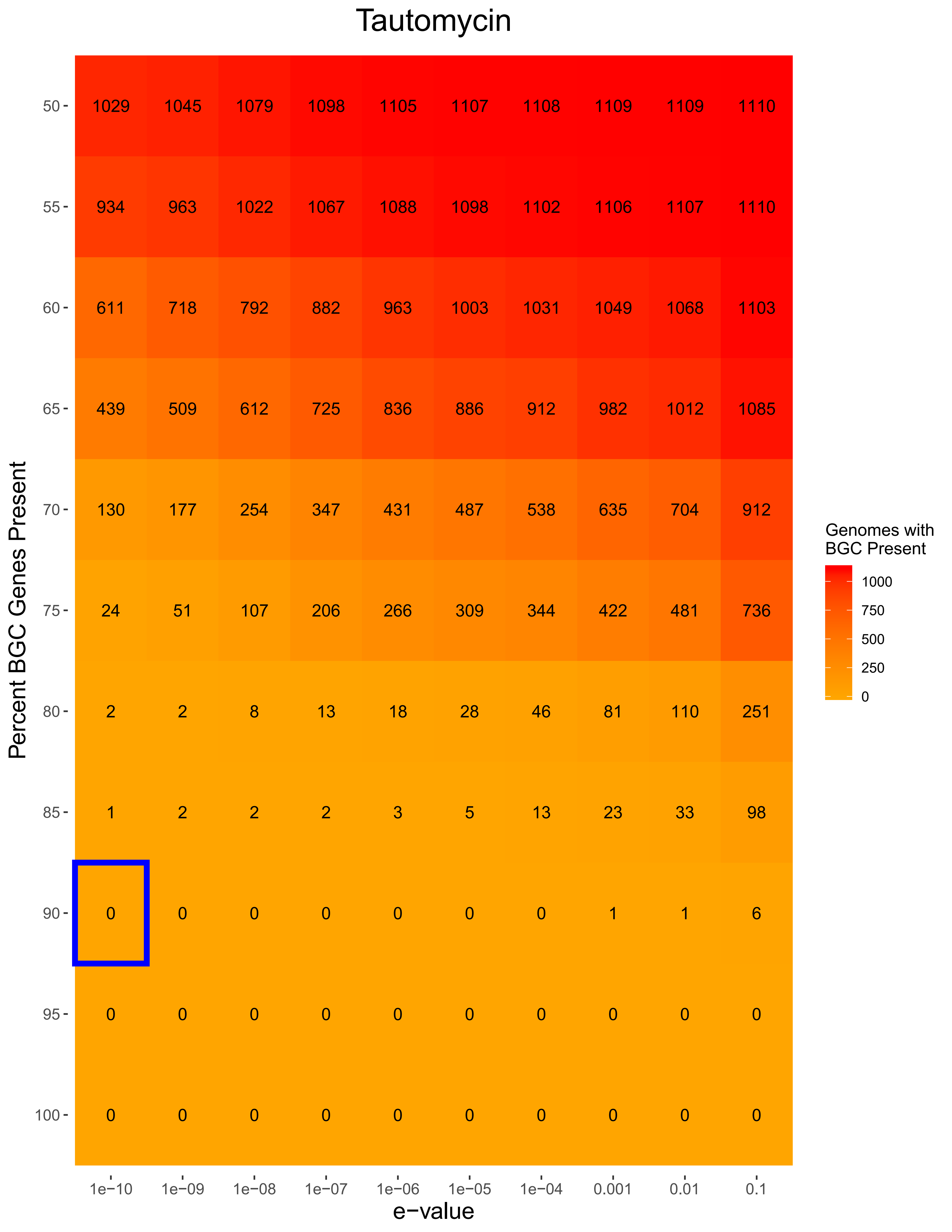
Tautomycin
Percent BGC Genes Present
e−value
1e−10
1e−09
1e−08
1e−07
1e−06
1e−05
1e−04
0.001
0.01
0.1
50
55
60
65
70
75
80
85
90
95
100
1029 1045 1079 1098 1105 1107 1108 1109 1109 1110
934 963 1022 1067 1088 1098 1102 1106 1107 1110
611 718 792 882 963 1003 1031 1049 1068 1103
439 509 612 725 836 886 912 982 1012 1085
130 177 254 347 431 487 538 635 704 912
24 51 107 206 266 309 344 422 481 736
2 2 8 13 18 28 46 81 110 251
1 2 2 2 3 5 13 23 33 98
0 0 0 0 0 0 0 1 1 6
0 0 0 0 0 0 0 0 0 0
0 0 0 0 0 0 0 0 0 0
Genomes with
BGC Present
1000
750
500
250
0

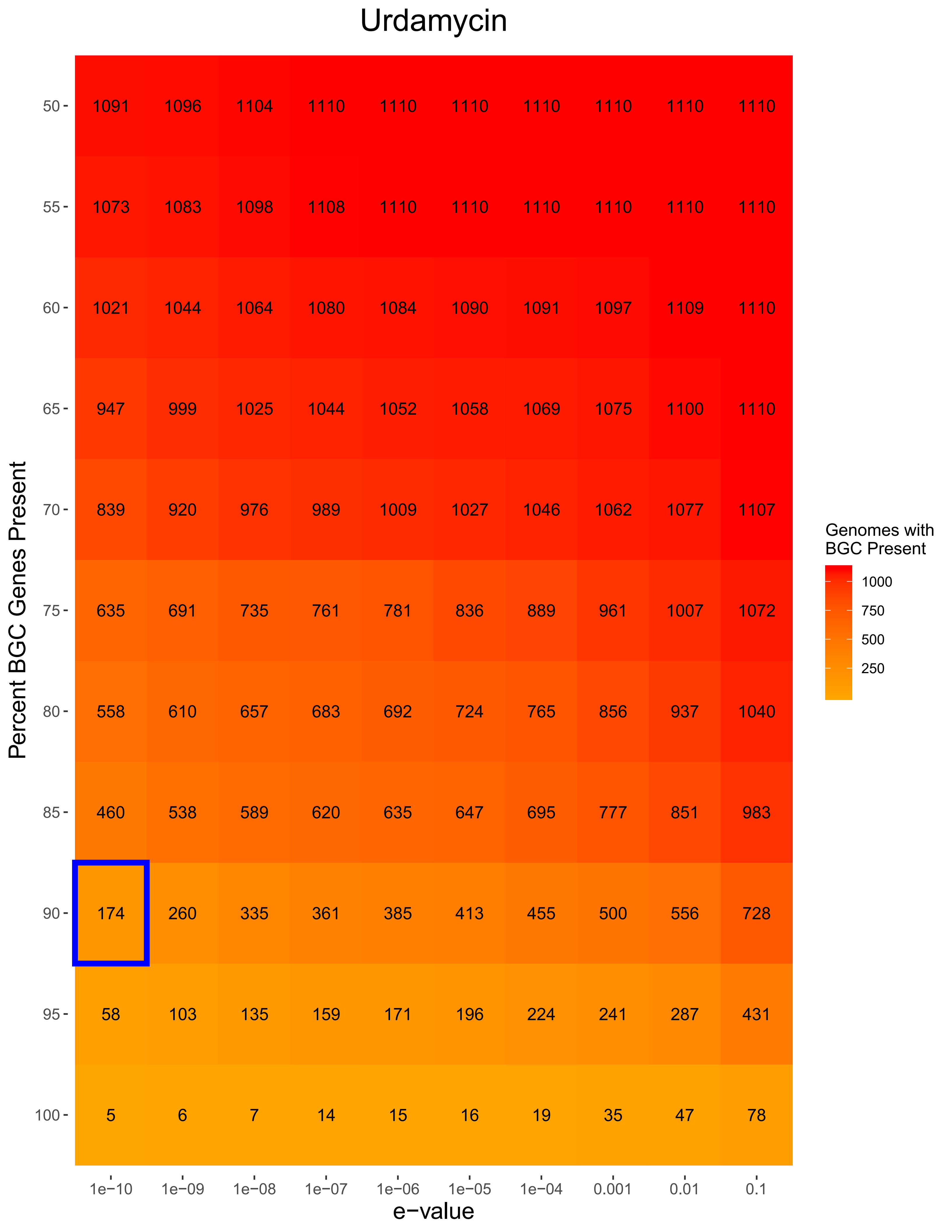
Urdamycin
Percent BGC Genes Present
e−value
Genomes with BGC Present
1000
750
500
250
50 55 60 65 70 75 80 85 90 95 100
1e−10 1e−09 1e−08 1e−07 1e−06 1e−05 1e−04 0.001 0.01 0.1
1091 1096 1104 1110 1110 1110 1110 1110 1110 1110
1073 1083 1098 1108 1110 1110 1110 1110 1110 1110
1021 1044 1064 1080 1084 1090 1091 1097 1109 1110
947 999 1025 1044 1052 1058 1069 1075 1100 1110
839 920 976 989 1009 1027 1046 1062 1077 1107
635 691 735 761 781 836 889 961 1007 1072
558 610 657 683 692 724 765 856 937 1040
460 538 589 620 635 647 695 777 851 983
174 260 335 361 385 413 455 500 556 728
58 103 135 159 171 196 224 241 287 431
5 6 7 14 15 16 19 35 47 78

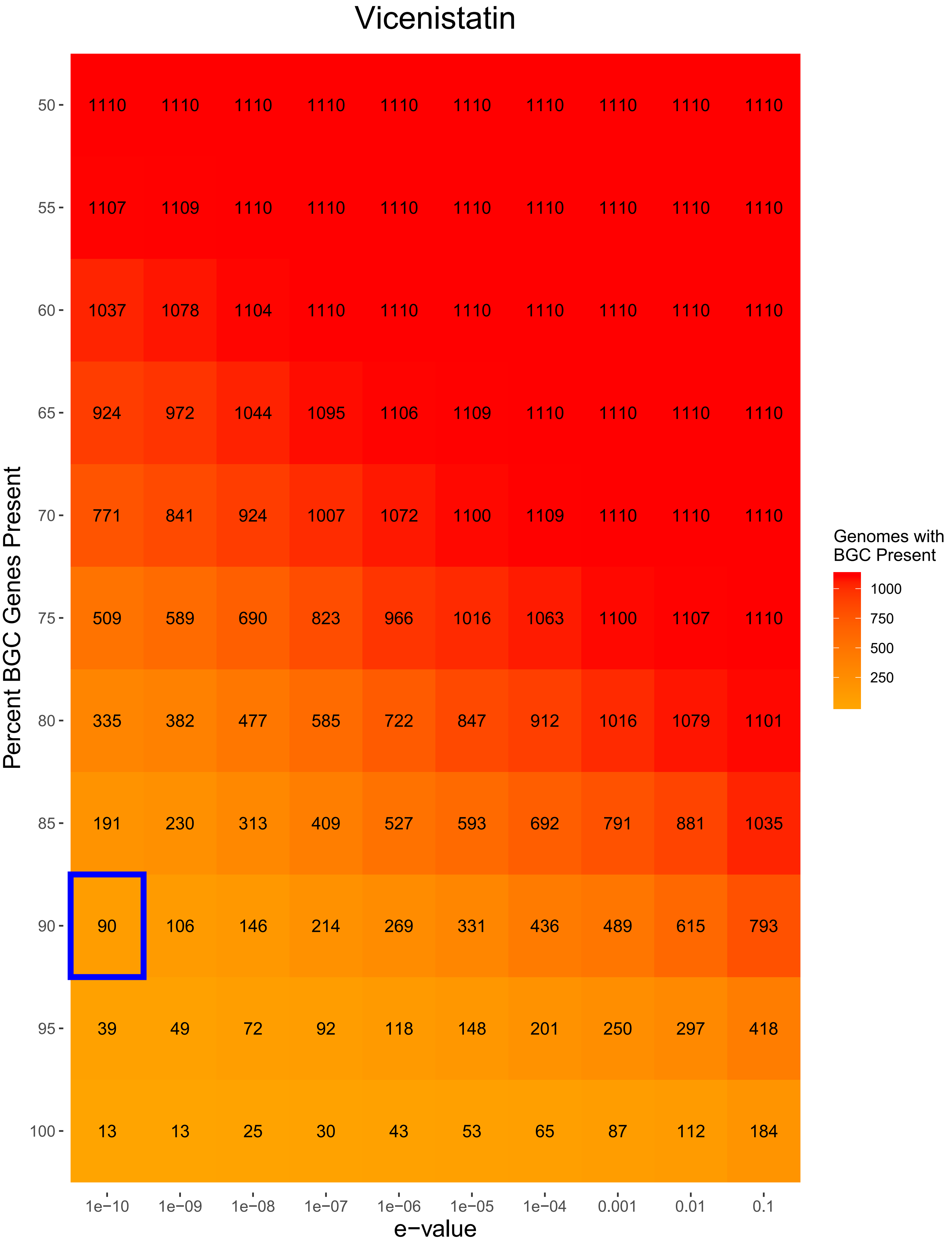

Vicenistatin
Percent BGC Genes Present
e−value
Genomes with BGC Present
50
55
60
65
70
75
80
85
90
95
100
1e−10
1e−09
1e−08
1e−07
1e−06
1e−05
1e−04
0.001
0.01
0.1
1000
750
500
250
1110 1110 1110 1110 1110 1110 1110 1110 1110 1110
1107 1109 1110 1110 1110 1110 1110 1110 1110 1110
1037 1078 1104 1110 1110 1110 1110 1110 1110 1110
924 972 1044 1095 1106 1109 1110 1110 1110 1110
771 841 924 1007 1072 1100 1109 1110 1110 1110
509 589 690 823 966 1016 1063 1100 1107 1110
335 382 477 585 722 847 912 1016 1079 1101
191 230 313 409 527 593 692 791 881 1035
90 106 146 214 269 331 436 489 615 793
39 49 72 92 118 148 201 250 297 418
13 13 25 30 43 53 65 87 112 184
